# The *Prrx1eGFP* Mouse Labels the Periosteum During Development and a Subpopulation of Osteogenic Periosteal Cells in the Adult

**DOI:** 10.1002/jbm4.10707

**Published:** 2022-12-14

**Authors:** Sarah Brown, Saif Malik, Maria Aljammal, Aine O'Flynn, Carl Hobbs, Mittal Shah, Scott J Roberts, Malcolm PO Logan

**Affiliations:** ^1^ Randall Centre for Cell and Molecular Biophysics King's College London London UK; ^2^ UCB Pharma Slough UK; ^3^ Department of Comparative Biomedical Sciences Royal Veterinary College London UK

**Keywords:** AGING, BONE, DEVELOPMENT, PERIOSTEUM, *Prrx1*, STEM CELL

## Abstract

The identity of the cells that form the periosteum during development is controversial with current dogma suggesting these are derived from a Sox9‐positive progenitor. Herein, we characterize a newly created *Prrx1eGFP* reporter transgenic mouse line during limb formation and postnatally. Interestingly, in the embryo *Prrx1eGFP*‐labeled cells become restricted around the Sox9‐positive cartilage anlage without themselves becoming Sox9‐positive. In the adult, the *Prrx1eGFP* transgene live labels a subpopulation of cells within the periosteum that are enriched at specific sites, and this population is diminished in aged mice. The green fluorescent protein (GFP)‐labeled subpopulation can be isolated using fluorescence‐activated cell sorting (FACS) and represents approximately 8% of all isolated periosteal cells. The GFP‐labeled subpopulation is significantly more osteogenic than unlabeled, GFP‐negative periosteal cells. In addition, the osteogenic and chondrogenic capacity of periosteal cells in vitro can be extended with the addition of fibroblast growth factor (FGF) to the expansion media. We provide evidence to suggest that osteoblasts contributing to cortical bone formation in the embryo originate from *Prrx1eGFP*‐positive cells within the perichondrium, which possibly piggyback on invading vascular cells and secrete new bone matrix. In summary, the *Prrx1eGFP* mouse is a powerful tool to visualize and isolate periosteal cells and to quantify their properties in the embryo and adult. © 2022 The Authors. *JBMR Plus* published by Wiley Periodicals LLC on behalf of American Society for Bone and Mineral Research.

## Introduction

Bone is a dynamic tissue that undergoes constant remodeling and repair throughout life; however, the identity of the stem cells that sit at the apex of the hierarchy that facilitates these processes has remained elusive. Current research on the biology of skeletal stem cells has concentrated on bone marrow stromal cells (BMSCs), which exist as perivascular cells and form the niche for hematopoietic stem cells (HSCs). These BMSCs show multipotency in vitro through differentiation toward adipocytes, chondrocytes, and osteoblasts when exposed to permissive culture conditions, however, limited evidence exists to suggest that they can form these cell types in vivo.^(^ ^1^, ^2^, ^3^ ^)^ Conversely, several studies have demonstrated the periosteum as a potential source of skeletal stem cells in vivo because of its fundamental role in providing osteoblasts and chondrocytes that contribute to appositional bone growth and fracture repair.^(^ ^4^, ^5^, ^6^ ^)^

The periosteum envelops the outer surface of all bones, except the regions covered by articular cartilage, and consists of an outer fibrous layer and inner cambium layer.^(^ ^7^, ^8^, ^9^ ^)^ The thicker, outer fibrous layer lies adjacent to surrounding soft tissue and muscle and contains relatively few cells. In contrast, the thinner, internal cambium layer directly opposes the outer surface of bone, and it is this layer that is believed to serve as a reservoir for stem cells capable of differentiating into chondrogenic and osteogenic lineages required for bone growth and repair.^(^ ^10^ ^)^ After trauma, the periosteum produces the cells of the cartilage callus that are ultimately remodeled to form bone in a process that, under normal conditions, results in remarkably efficient “scar‐free” healing.^(^ ^11^ ^)^ Cells resident within the periosteum can contribute to bone repair by recapitulating features of bone development. After an initial burst of proliferation, cells undergo chondrogenic differentiation to initiate endochondral bone formation.^(^ ^12^, ^13^, ^14^ ^)^

A number of molecular markers, such as Cathepsin K, have been proposed for the stem/progenitor cells resident within the adult periosteum;^(^ ^15^ ^)^ however, analysis of the cellular composition of the forming periosteum during early developmental stages is lacking. The absence of robust reporters that can be used to identify cells associated with periosteum formation has impeded research in this area of skeletal biology. Indeed, longitudinal analysis of periosteal tissue during endochondral development of skeletal elements in the appendicular skeleton is lacking.

*Prrx1* and the closely related *Prrx2* gene are involved in the formation and growth of chondrogenic and osteogenic precursors.^(^ ^16^, ^17^ ^)^ During development, *Prrx1* and *Prrx2* are initially broadly expressed in the limb buds, including the preskeletogenic condensations, and subsequently become restricted to the periosteum.^(^ ^18^, ^19^, ^20^, ^21^ ^)^ This poses the possibility that the progenitor cells that contribute to embryonic skeletal elements through endochondral ossification are sequestered postnatally for bone repair and homeostasis. This would be analogous to the Pax3/Pax7‐positive satellite cells that are sequestered within muscles as tissue‐resident stem cells that contribute to postnatal muscle growth and repair.^(^ ^22^ ^)^

Identification of a *Prrx1* regulatory element^(^ ^23^ ^)^ enabled development of transgenic lines, including *Prrx1Cre* ^(^ ^24^ ^)^ and *Prrx1CreER‐GFP*.^(^ ^25^ ^)^ In *Prrx1Cre* mice, transgene activity is detectable throughout the limb mesenchyme, which will give rise to skeletal elements, tendons, and connective tissues.^(^ ^24^ ^)^ Limb muscles form from progenitor cells originating in the somites adjacent to the limb bud;^(^ ^26^ ^)^ however, after these precursors enter the limb, they activate expression of the *Prrx1Cre* transgene. Thus, limb muscles at embryonic and adult stages are targeted by *Prrx1Cre* activity.^(^ ^24^, ^27^ ^)^ *Prrx1CreER‐GFP*‐labeled periosteal cells were found to have enhanced osteogenic and chondrogenic potential and contributed to fracture callus formation in vivo. However, green fluorescent protein (GFP) expression in the *Prrx1CreER‐GFP* transgenic is weak, making cell isolation and detection challenging.^(^ ^25^, ^28^ ^)^ Prrx1^+^ cells have also been identified in the calvaria in addition to the long bones of the limbs.^(^ ^29^ ^)^ Prrx1^+^ cells have also been detected in the dermis postnatally, labeling a small subset of cells within dermal perivascular and hair follicle niches.^(^ ^30^ ^)^

Here, we report a *Prrx1eGFP* transgenic mouse model that allows the stage‐specific identification and characterization of periosteal cells during development and in the adult. Unlike previous tamoxifen‐inducible lines (eg, Prrx1‐creER‐IRES‐eGFP), this is the first line that directly drives “live” GFP expression. This allows for a “real‐time” readout of the Prrx1 transgene activity throughout embryonic development and into adulthood.

Herein, we report that during embryonic stages, cells marked by *Prrx1* become gradually restricted to tissues around the cartilaginous anlage, which later becomes the perichondrium. In the adult, when isolated, periosteal cells age in vitro, becoming less osteogenic with each subsequent passage. In vivo, *Prrx1eGFP*‐labeled cells are enriched in areas of loading and are reduced in aged mice, suggesting *Prrx1eGFP* is labeling a stem/progenitor cell population within the periosteum. These data reveal the association of *Prrx1*‐expressing cells to the forming periosteum and demonstrate the *Prrx1eGFP* transgenic mouse as a powerful tool for studying periosteal biology in both in the embryo and adult.

## Materials and Methods

### Transgenic mice

The *Prrx1eGFP* transgenic construct was generated by cloning the coding sequence of enhanced GFP (eGFP) into the *Prrx1* transgenic backbone described previously.^(^ ^24^ ^)^ Transgenic founders were produced via pronuclear injection at the Procedural Services Section, NIMR. Several founder animals were identified by positive PCR genotyping from ear biopsy. Germline transmission and the efficacy of the reporter were tested by crossing F0 males or F1 males from F0 females to CD1 females and analyzing GFP expression in embryonic limb buds from litters harvested at E10.5. The founder that produced the brightest GFP expression in the limbs as judged by visual inspection under episcopic fluorescent illumination was chosen and crossed onto CD1 background. Germline transmission and observed GFP expression in this line has remained stable for >5 years maintained as a heterozygous line. Mouse embryos were staged according to Kaufman.^(^ ^31^ ^)^ Noon on the day a vaginal plug was observed was taken to be E0.5 days of development. Mice were euthanized by cervical dislocation. Mouse genotyping was performed by PCR using primers 5'TGCAGTGCTTCAGCCGCTAC (GFP Fwd) and 5'CCAGCAGGACCATGTGATC (GFP Rev). Genetically altered mouse lines were generated and maintained under appropriate home office license authority and were reviewed and approved by local (King's College London) Ethical Review Panel (ERP).

### Immunohistochemistry, immunocytochemistry, and histology

Dissected limbs were fixed in 4% PFA overnight at 4°C and decalcified in 10% EDTA for 2 weeks before cryopreservation with 30% sucrose. Specimens were embedded in OCT and frozen on dry ice before 8 μm sections were cut at −21°C on a Leica (Wetzlar, Germany) CM1950 cryostat.

Immunohistochemical staining was performed by washing the slides with phosphate‐buffered saline (PBS) for 5 minutes, followed by PBS/0.1% Triton X‐100 for 5 minutes at room temperature. Slides were then blocked with 1 mL of blocking solution (10% goat or donkey serum [Gibco, Thermo Fisher Scientific, Waltham, MA, USA], 0.1% Triton X‐100) for 1 hour at room temperature. Primary antibody (200 μL diluted in blocking solution, dilution listed below) was used per slide and incubated at 4°C overnight. Slides were washed five times in an hour with PBS/0.1% Triton X‐100. Secondary antibody (200 μL diluted in blocking solution, dilution listed below) was used per slide and incubated at room temperature for 2 hours in the dark. Slides were washed in the dark five times with PBS + 0.1% Triton X‐100, once with PBS, and counterstained with DAPI (diluted 1:15,000 in PBS) for 4 minutes. Images were taken using a Leica Dmi8 or Nikon (Tokyo, Japan) A1 confocal. Primary and secondary antibodies were as follows: chicken‐polyclonal‐anti‐GFP (1:500, ab13970), rabbit‐polyclonal‐anti‐periostin (1:100, ab14041), SOX9 (1:10, NL3075R), rabbit‐polyclonal‐anti‐Runx2 (1:500, ab23981). Secondary Antibodies (all from Abcam, Cambridge, UK): goat‐anti‐chicken‐alexa‐fluor‐488 (1:500, ab11039), goat‐anti‐rabbit‐alexa‐fluor‐594 (1:500, ab150080), goat‐anti‐rabbit‐alexa‐fluor‐647 (1:500, ab150079).

For histology, decalcified specimens were embedded in Fibrowax (VWR Chemicals, Avantor, Radnor, PA, USA), and 6 μm sections were cut using a Reichert‐Jung (Leica) Autocut 2010 Microtome. Slides were dewaxed in xylene for 10 minutes and then briefly washed in absolute alcohol. Slides were then stained using Mayer's hemalum before being placed in either Van Geison's solution for HVG staining or hemalum (Gill's No. 1) and then 0.5% eosin for hematoxylin and eosin (H&E) staining. Anti‐GFP immunostaining was performed after dewaxing by incubating slides in 3% hydrogen peroxide for 10 minutes before washing with tap water and blocking for 10 minutes in 2% bovine serum albumin (BSA) in 1× Tris‐buffered saline (TBS) and 10% azide, pH 7.6. Slides were incubated with anti‐GFP primary antibody (1:500 dilution, Rockland, Pottstown, PA, USA) at room temperature and then washed in 1× TBS for 10 minutes. Anti‐goat secondary biotinylated antibody (1:300, Vector Laboratories, Burlingame, CA, USA) was added for 60 minutes at room temperature. An amount of 1 μL streptavidin and 1 μL biotinylated horseradish peroxidase (HRP) were mixed into 100 μL 1× TBS and left to conjugate for 30 minutes to form the ABC peroxidase complex. Slides were washed twice in 1× TBS for 5 minutes before incubating in the ABC peroxidase complex for 30 minutes at room temperature. Slides were washed twice in 1× TBS for 5 minutes on a shaker before developing for 10 minutes in the DAB solution. Slides were then washed under a running tap for 5 minutes before counterstaining with hemalum for 2 minutes and then washed again under running tap water until the water ran clear. Each slide was examined using a light microscope to check optimal staining intensity. Slides were then rinsed briefly in distilled water, blotted gently, and left to dry. After drying, slides were rinsed briefly in 100% alcohol before rinsing in xylene twice for 5 minutes and finally mounting with DPX mounting medium (Sigma‐Aldrich, St. Louis, MO, USA).

Periosteal thickness measurements were taken using annotation tools on Lecia LAS X software. Sections were chosen that had both regions of muscle insertion and muscle origin in the same cross section. Four serial sections were taken from the tibial crest region of four *Prrx1eGFP* transgenic mice. Periosteal thickness and GFP content data were analyzed using GraphPad Prism (GraphPad, La Jolla, CA, USA), and statistics were carried out using multiple unpaired *t* tests (Holm–Sidak method).

### Optical projection tomography (OPT)

Whole embryos were washed in PBS/0.1% Triton X‐100 for 30 minutes before staining with 500 μL GFP Tag Polyclonal Antibody, Alexa Fluor 488 conjugate (A21311, Life Technologies, Invitrogen, Carlsbad, CA, USA), diluted 1:400 with blocking buffer (PBS with 1% BSA, 0.15% glycine, 0.1% Triton X‐100) for at least 7 days at 4°C. Specimens were then washed three times in 0.1% Triton X‐100 for 5 minutes each time at room temperature, and once again overnight at 4°C. Specimens were post‐fixed in 4% PFA in PBT (0.1% Tween in PBS) for 30 minutes at room temperature and washed three times, 5 minutes each time, in PBT at room temperature. OPT was used to create 3D images of fluorescent‐stained embryonic and postnatal *Prrx1eGFP* tissue following the methods outlined previously.^(^ ^32^, ^33^ ^)^ Fiji/Image J^(^ ^34^ ^)^ and Osirix software (Pixmeo SARL) was used to create 3D images and videos.

### Periosteal cell isolation

To isolate murine periosteal‐derived cells (PDC), 6‐ to 10‐week‐old *Prrx1eGFP* mice were culled and cells were isolated and cultured using methods adapted from Duchamp de Lageneste and colleagues.^(^ ^5^ ^)^ Culled mice were cleaned with 70% ethanol, hindlimbs were skinned, and tibias and femur were removed and kept hydrated in PBS. In a flow cabinet, femurs and tibias were cleaned thoroughly with clean tissues (Kimberly‐Clark, Kimcare Interfold Medical Wipes, Fisher Scientific, Pittsburgh, PA, USA) to remove any remaining muscle and connective tissue and transferred to a clean 10 cm dish with warmed media (minimum essential media [MEM] containing 10% fetal bovine serum [FBS], 1% L‐glutamine, 1% penicillin streptomycin [P/S] [Gibco, 15140122]). To prevent removal of cells at the epiphyses, these were submerged in 5% low melting point agarose (Sigma, A9414). After the agarose solidified, PDCs were isolated by a 1‐hour collagenase dispase digest (3 mg/mL collagenase [Gibco, 17104019] and 4 mg/mL dispase [Gibco, 17105041] in MEM [filtered through a 0.45 μm syringe filter]) at 37°C. The cell suspension was passed through a 70 μm nylon mesh (BD Falcon, BD Biosciences, San Jose, CA, USA) into a 50 mL falcon tube. Growth medium was added to the cell suspension up to 50 mL and centrifuged at 700 g for 10 minutes. The cell pellet was resuspended in growth medium and the total‐cell yield of 5 bones was plated into 1 well of a 6‐well plate. Growth media was changed every 2 days, and cells were passaged at a 1:3 ratio when they reached 80% to 90% confluence. Basic FGF was added to growth media (Gibco, PMG0034, final concentration 5 ng/mL) to facilitate the expansion of mPDCs.

### Osteogenic differentiation assays

MC3T3‐E1 (clone 14) or PDCs were plated at a density of 10,000 cells/cm^2^ in a 24‐well plate and cultured for 21 days in osteogenic conditions (50 μg/mL ascorbic‐2‐phosphate [Sigma A8960‐5G] 5 mM β‐glycerophosphate [Sigma, G6251‐10G]) before staining with 1% Alizarin Red, as previously described.^(^ ^5^ ^)^ To quantify staining, 500 μL 10% cetylpyridinium chloride (Sigma‐Aldrich) was added to each well for 1 hour at 37°C with shaking. Serial dilution colorimetric assays were set up and absorbance was read at 540 nm on a SpectroMax i3x plate reader with SoftMax Pro version 6.0 software (Molecular Devices, Wokingham, UK). Data were analyzed using GraphPad Prism (9.0.0), using multiple comparisons 2‐way ANOVA.

### Chondrogenic differentiation assays

PDCs were pelleted and resuspended to a concentration of 20 × 10^6^ cells/mL, plated in 10 μL micromasses, cultured for 7 days (50 μg/mL ascorbic‐2‐phosphate [Sigma A8960‐5G], 10 ng/mL recombinant mouse transforming growth factor [TGF]‐ß1 [PeproTech, Rocky Hill, NJ, USA, 100–21]) and stained with Alcian Blue as previously described.^(^ ^5^ ^)^

### FACS analysis

After periosteal cell isolation, cells were sorted by fluorescence‐activated cell sorting with an Aria II Flow sorter at the NIHR Guy's and St Thomas' BRC Flow Cytometry Platform, before plating (P0). Wild‐type samples were used as controls to ensure correct gating of *Prrx1eGFP* transgenic samples. Cells were pooled from five transgenic mice resulting in ~33,000 to 60,000 GFP‐positive cells, and 420,000 to 800,000 GFP‐negative. An amount of 1 μL 10 mM DAPI was added to cell samples before sorting and used as a dead cell marker to exclude dead cells from sorting. Cell viability post‐FACS was determined using trypan blue (Sigma, T8154‐20ML). A total of 10 μL cell suspension was mixed with 10 μL 0.4% trypan blue solution (1:1 ratio), and cells were counted using a hemocytometer. Cells were then seeded into 1 well of a 6‐well plate per condition.

## Results

### The *Prrx1eGFP* transgenic line is a live cell marker of a subset of periosteal cells

**Fig. 1:**
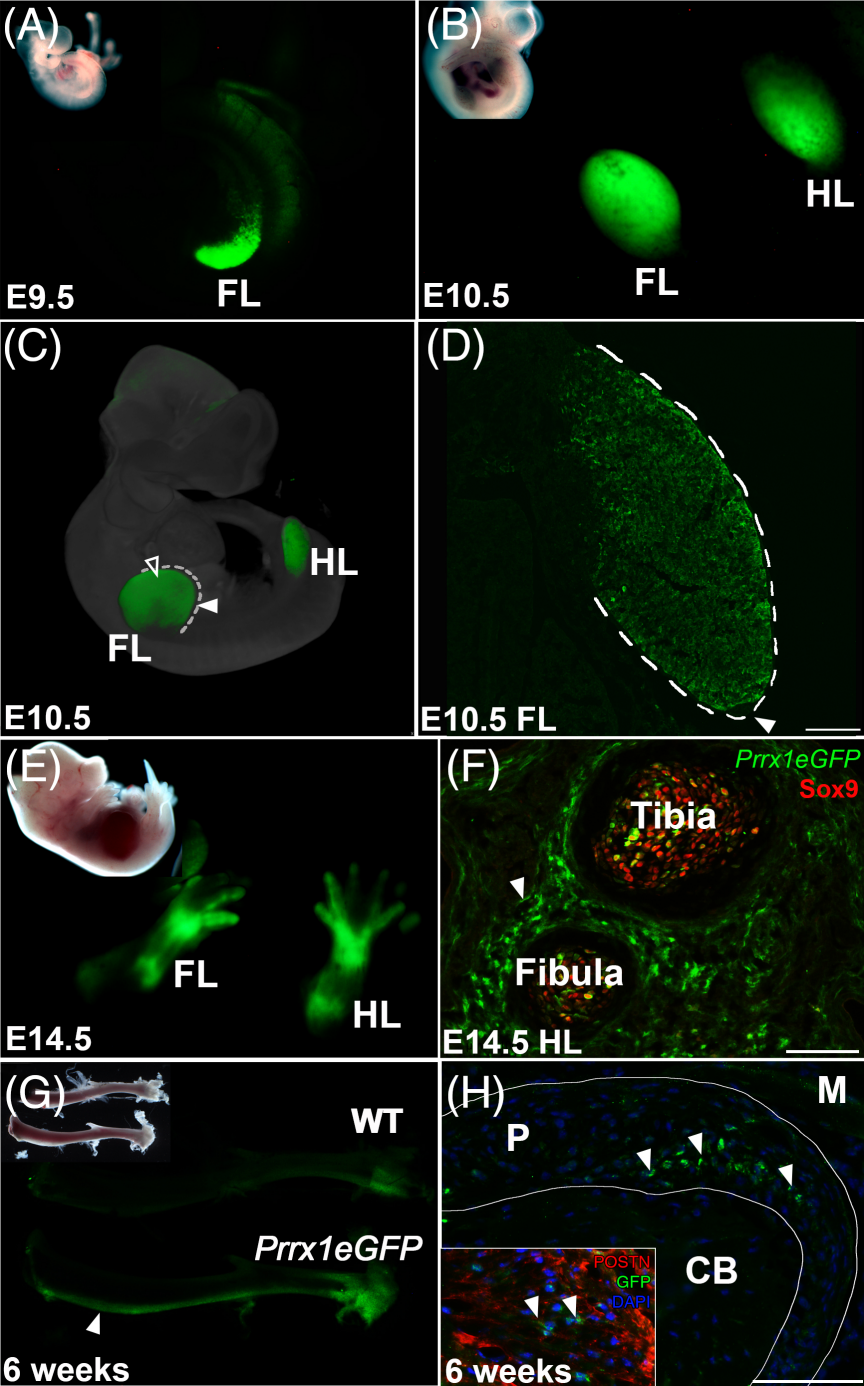
*Prrx1eGFP* is a live cell marker of the early limb bud mesenchyme and later becomes restricted to a subset of periosteal cells by late embryonic stages. (*A*) Live GFP expression in an E9.5 *Prrx1eGFP* forelimb. Inset panel is bright‐field image of the same embryo. (*B*) Live GFP expression in an E10.5 *Prrx1eGFP* forelimb and hindlimb. Inset panel is bright‐field image of the same embryo. (*C*) OPT composite image showing GFP labeling in the forelimb and hindlimb mesenchyme (hollow arrowhead). GFP is absent from the surrounding ectoderm (solid arrowhead). (*D*) Section IHC for GFP on an E10.5 forelimb stain showing GFP expression in the mesenchyme but not the surrounding ectoderm (outlined by the white lines) or in the apical ectodermal ridge (AER) (white arrow). Scale bar = 100 μm. (*E*) E14.5 *Prrx1eGFP* embryo showing live GFP expression in the developing limbs. Inset panel is bright‐field image of the same embryo. (*F*) Section IHC for GFP (green) and SOX9 (red) on an E14.5 forelimb, 20× magnification. Scale bar = 100 μm. GFP is enriched in a ring of cells surrounding the Sox9‐positive cartilage anlagen. Some GFP‐positive cells are observed in the cartilage anlagen at this stage. (*G*) Live GFP expression in a 6‐week‐old *Prrx1eGFP* tibia. (*H*) Section IHC of a 6‐week‐old tibia. Scale bar = 75 μm. A subpopulation of cells within the periosteum (outlined with white lines) are GFP‐positive (white arrows). GFP‐positive cells are not observed in cortical bone. Inset of a serial section stained with the periosteal marker, periostin (red), GFP (green), and DAPI nuclear stain (blue). FL = forelimb; HL = hindlimb; WT = wild‐type; P = periosteum; CB = cortical bone.

To examine the distribution of GFP expression in the *Prrx1eGFP* transgenic during embryonic stages, we analyzed embryos that spanned key stages in limb development from E9.5 to E14.5 (Fig. 1*A–F*). Using episcopic fluorescence illumination on unfixed specimens, robust GFP expression was detected in the forelimb bud at E9.5 (Fig. 1*A*). At this stage, no GFP expression was detected in the hindlimb. By E10.5, however, GFP was observed in both the forelimb and hindlimb (Fig. 1*B*). To study the distribution of GFP‐positive cells at cellular resolution, samples were analyzed using immunohistochemistry (IHC). Optical projection tomography (OPT) scanning of stained embryos reveled GFP expression was restricted to limb mesenchyme cells and is not present in the overlying ectoderm (Fig. 1*C*, arrowheads). This distribution is confirmed by IHC staining of transverse sections through the limb and absence of staining in the apical ectodermal ridge (AER) is clearly visible (Fig. 1*D*, white arrowhead). Although GFP expression is present throughout the limb bud, the intensity of staining in cells varies in a “salt‐and‐pepper” pattern. By E14.5, GFP is no longer broadly expressed throughout cells of the limb mesenchyme (Fig. 1*E*) and is becoming restricted to regions surrounding the forming bones (Fig. 1*F*, white arrow), although some GFP‐positive cells remain visible within the cartilaginous template of the forming bone. Postnatally, at 6 weeks of age, GFP expression can be detected under episcopic fluorescence illumination and the bones appear green, whereas no GFP expression can be observed in wild‐type animals (Fig. 1*G*). Endogenous GFP expression from the *Prrx1eGFP* transgene is difficult to detect at single‐cell resolution but immunohistochemical staining of sections of 6‐week‐old tibia demonstrate that the GFP‐positive cells are restricted to a minor subpopulation of cells within the periosteum (Fig. 1*H*). Labeling of the surrounding environment with the periosteal extracellular matrix protein, periostin (Postn), confirms the presence of GFP‐positive cells within the periosteal layer (Fig. 1*H*, inset). There is minimal *Prrx1eGFP* expression detected in the growth plate at this stage (Supplemental Fig. S1), whereas at earlier embryonic stages, *Prrx1eGFP* can be detected in a small population of proliferative and hypertrophic chondrocytes in the growth plate. We also detect live episcopic GFP expression in cranial facial regions of the *Prrx1eGFP* transgenic from E11.5 onward (seen in Fig. 1*E* at E14.5).

### *Prrx1eGFP* ‐labeled cells from perichondrium are closely associated with the emerging osteoprogenitor pool, which are in turn adjacent to endothelial cells

**Fig. 2:**
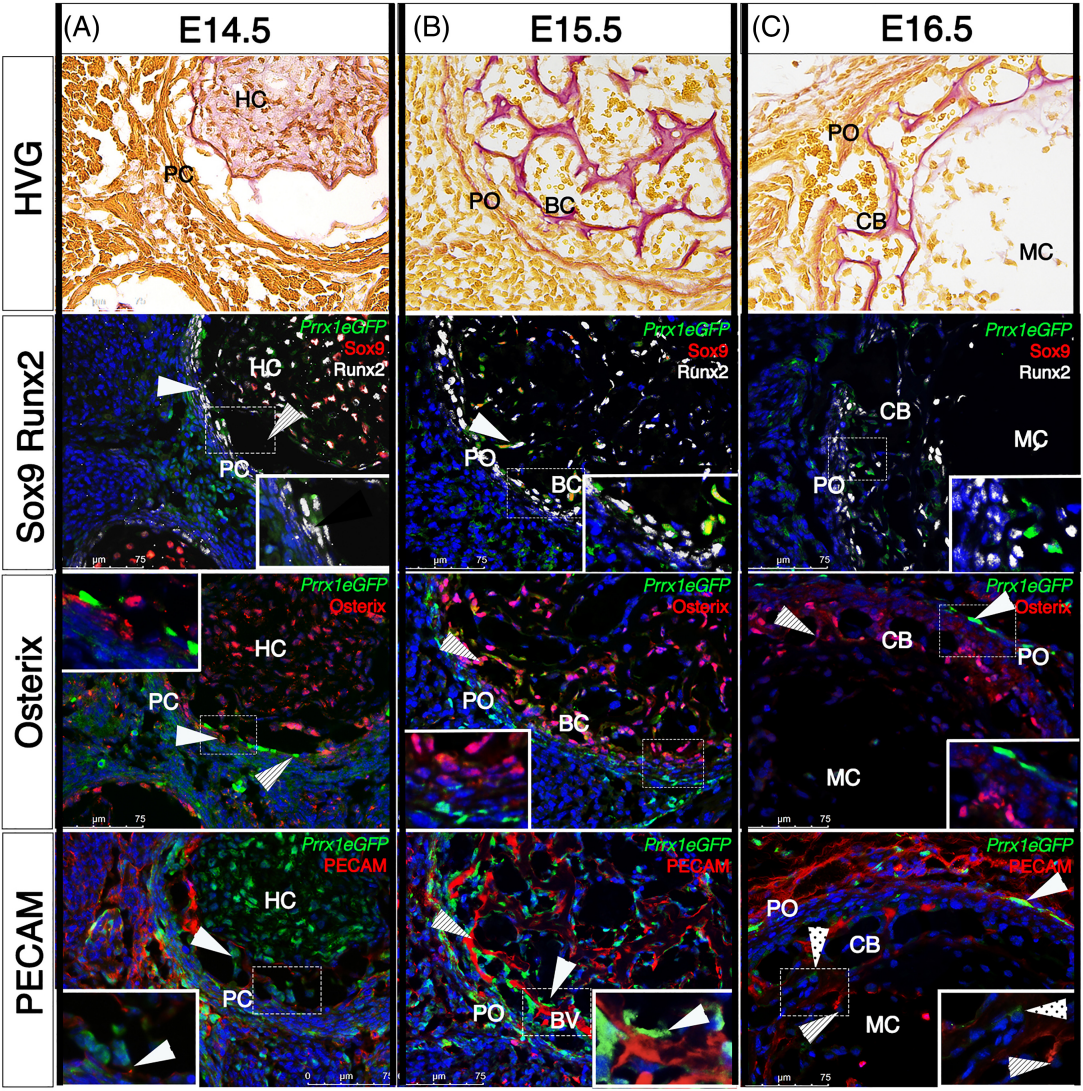
*Prrx1eGFP* GFP‐expressing cells of the perichondrium are closely associated with the formation of the bone collar and with invading blood vessels. Serial sections of *Prrx1eGFP* hindlimbs from E14.5 (*A*), E15.5 (*B*), and E16.5 (*C*) stained alternately with HVG, Sox9, and Runx2, Osterix, and PECAM. All fluorescence‐stained sections were also stained for GFP and DAPI. Scale bar = 75 μm. *n* = 3. (*A*) The mesenchymal condensation retracts away from the Runx2‐positive perichondrium (white arrowhead), creating a space (dashed arrowhead). *Prrx1eGFP/*Runx2‐double‐positive cells at the inner surface of the perichondrium begin to invade this space (inset). Vascular cells occupy the space (*A*, *B*, dashed arrowhead) between the cartilage and periosteum and *Prrx1eGFP*‐labeled cells are found in close association with PECAM‐positive endothelial cells (arrowheads and inset) and express Runx2 (arrowhead) and Osterix (dashed arrowhead). (*A–C*) The osteoid is secreted in a similar pattern forming the nascent bone collar. At E16.5 (*C*), the number of *Prrx1eGFP*‐labeled cells in the periosteum is becoming restricted (white arrowheads). Osterix‐labeled cells are found in association with blood vessels (dashed arrowheads). A few weak GFP‐positive cells are present within the forming cortical bone (dotted arrowhead). PC = perichondrium; PO = periosteum; HC = hypertrophic chondrocytes; BC = bone collar; CB = cortical bone; MC = marrow cavity.

We used the restriction in expression of the *Prrx1eGFP* transgene in the forming limb to analyze the development of the periosteum and its contribution to bone formation in the embryo. Using immunohistochemical methods, we examined mid‐diaphyseal sections of E14.5–E16.5 tibia. We stained serial sections of specimens using Sox9, as a marker of the early chondrogenic lineage, Runx2 and its downstream target Osterix (Osx), as markers of the osteogenic lineage and hypertrophic chondrocytes, PECAM as a marker of the invading endothelial cells that form blood vessels, and Postn as a marker of the developing periosteum (Fig. 2 and Supplemental Fig. S2). An HVG‐stained series was also included to indicate the progress of periosteal development and bone deposition.

Sox9 staining confirms the mesenchymal condensation is cartilaginous at E14.5, and through the compaction process, these cells become distant from the surrounding perichondrium (Fig. 2*A*, dashed arrow). The central Sox9‐positive cells also express Runx2 and Osx, indicating they are hypertrophic chondrocytes (HC). By E15.5, Sox9 staining has markedly reduced as the bone marrow cavity develops (Fig. 2*B*), and by E16.5, the marrow cavity is devoid of all Sox9‐positive cells (Fig. 2*C*).

At E14.5, *Prrx1eGFP* transgene expression is enriched in the perichondrium (PC), which is Runx2‐positive, Sox9‐negative, suggesting perichondrial cells have osteogenic potential (Fig. 2*A*, inset). Serial sections show Osx‐positive cells, co‐labeled with *Prrx1eGFP* and Runx2, in the innermost layer of the perichondrium (Fig. 2*A*). By E15.5, HVG staining illustrates the beginnings of osteoid deposition and the formation of the bone collar (BC), and therefore the transition from perichondrium (PC) to periosteum (PO) (Fig. 2*B*). There is a distinct ring of *Prrx1eGFP*‐labeled cells, coinciding with the location of the periosteum, which are Runx2‐positive. Serial sections demonstrate Runx2‐Osterix‐double‐positive periosteal cells internal to Runx2‐positive‐Osx‐negative cells (Fig. 2*B*). This suggests a wave of osteogenic commitment from Runx2‐postive/Osx‐negative *Prrx1eGFP* labeled osteoblast progenitors in the periphery, to Runx2‐Osx double‐positive committed osteoblasts internally. The distribution of Osx‐positive committed osteoblasts is the same as the pattern of osteoid deposition observed after HVG staining, suggesting these cells are actively secreting the forming bone matrix (Fig. 2*B*). By E16.5, a subpopulation of cells within the periosteum are labeled by the *Prrx1eGFP* transgene (Fig. 2*C*), similar to what is found in the adult. These cells are Osx‐negative (Fig 2*C*, white arrowhead), suggesting they are not committed to the osteoblast lineage.

Blood vessels play an important role in regulating bone morphology.^(^ ^35^, ^36^ ^)^ Vascular endothelial cells guide the formation of a collagenous template upon which the bone matrix is deposited. Vessels become coated with collagen type I via the action of osteoblasts secreting directly onto endothelial cells forming the osteoid template that is subsequently mineralized to form bone.^(^ ^37^ ^)^ Using PECAM/CD31 (hereafter referred to only as PECAM) as a marker of vascular endothelial cells indicates that endothelial cell invasion begins at E14.5 (Fig. 2*A*) and develops into an intricate network of nascent blood vessels throughout the primary ossification center by E15.5 (Fig. 2*B*). *Prrx1eGFP*‐labeled cells expressing Runx2 and Osx are found in close association with invading endothelial cells (Fig. 2*C*, white arrowhead and inset). Taken together, these results suggest *Prrx1eGFP* cells of the perichondrium differentiate into Osx‐positive committed osteoblasts that contribute to the formation of the bone collar by remaining closely associated with invading endothelial cells and secreting new bone matrix.

Periostin shows poor specificity in labeling the developing periosteum in the embryo (Supplemental Fig. S2*A–C*). At E14.5, *Prrx1eGFP* is more effective at delineating the developing periosteum. By E15.5, periostin can be observed labeling the periosteum; however, there is still staining in the dermis and surrounding mesenchyme. This demonstrates that the *Prrx1eGFP* transgene can label the forming periosteum earlier and more robustly than periostin. Intriguingly, GFP staining is not distributed uniformly across sections, indicating localized enrichment of *Prrx1eGFP*‐labeled cells.

Cathepsin K is an enzyme involved in the resorption of bone and is a marker of osteoclasts. Recently, it has also been reported to be a marker of periosteal stem cells.^(^ ^15^ ^)^ During embryonic development, we observe Cathepsin K expression within hypertrophic chondrocytes at E14.5 (data not shown) and in a subpopulation of cells at the innermost layer of the periosteum, adjacent to the developing bone, at E16.5 (Supplemental Fig. S2*C*). However, we do not detect any overlap between *Prrx1eGFP*‐labeled cells in periosteum and Cathepsin K at any time point between E13.5–E18.5 (data not shown), indicating these are two separate cell populations.

### Histological staining can distinguish the outer fibrous layer and inner cambium of the periosteum in the adult

**Fig. 3:**
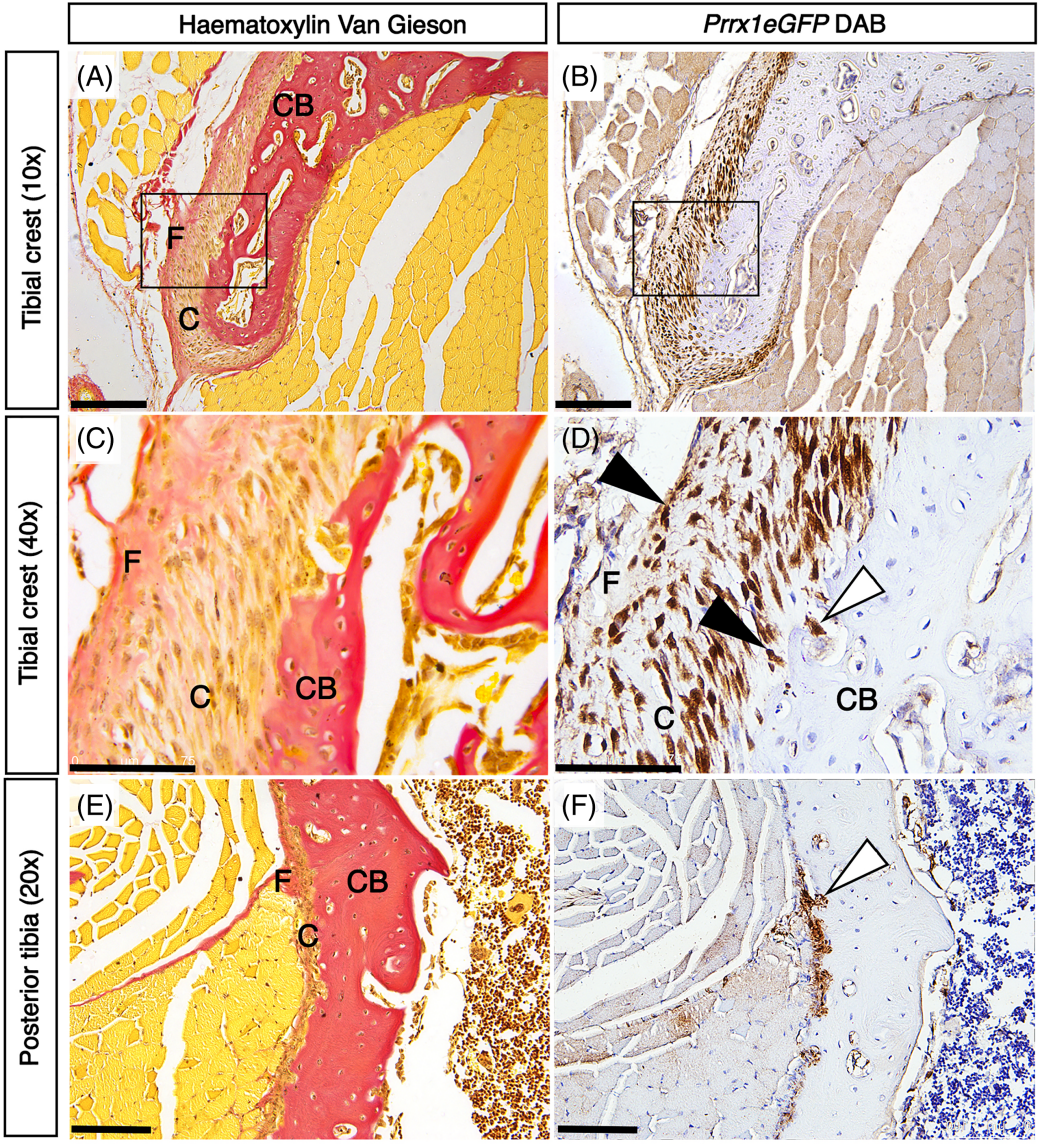
Comparative analysis with histological stains identify the location of *Prrx1eGFP*‐labeled periosteal cells. Serial cryosections of 6‐week‐old mouse hindlimbs, stained alternately with hematoxylin Van Gieson (*A*, *C*, *E*) and anti‐GFP antibody (*B*, *D*, *F*). 10× magnification of the tibial crest region (*A*, *B*). Scale bar = 200 μm. (*C*, *D*) 40× magnification of the boxed regions in *A*, *B*. Scale bar = 75 μm. 20× magnification of posterior tibia (*E*, *F*) Scale bar = 100 μm. Black arrowheads indicating GFP‐positive cells in both the cambium and fibrous layers. White arrowheads indicate GFP‐positive cells within cortical bone. F = fibrous; C = cambium layers; CB = cortical bone.

Fluorescent immunohistochemical staining for GFP on sections of bone does not show the surrounding bone and periosteum tissue histology. Therefore, we used a combination of a histological and immunohistochemical staining to confirm the anatomical location of the GFP‐positive cells in the adult mouse (Fig. 3). Hematoxylin and eosin staining labels the structure of the bone, muscle, and periosteum; however, the cambium and fibrous layers are not clearly distinguished from one another (Supplemental Fig. S3*A*). Toluidine blue, commonly used to highlight proteoglycans in cartilage and the fibrocartilaginous enthesis,^(^ ^38^, ^39^ ^)^ shows only weak staining in the periosteum (Supplemental Fig. S3).

**Fig. 4:**
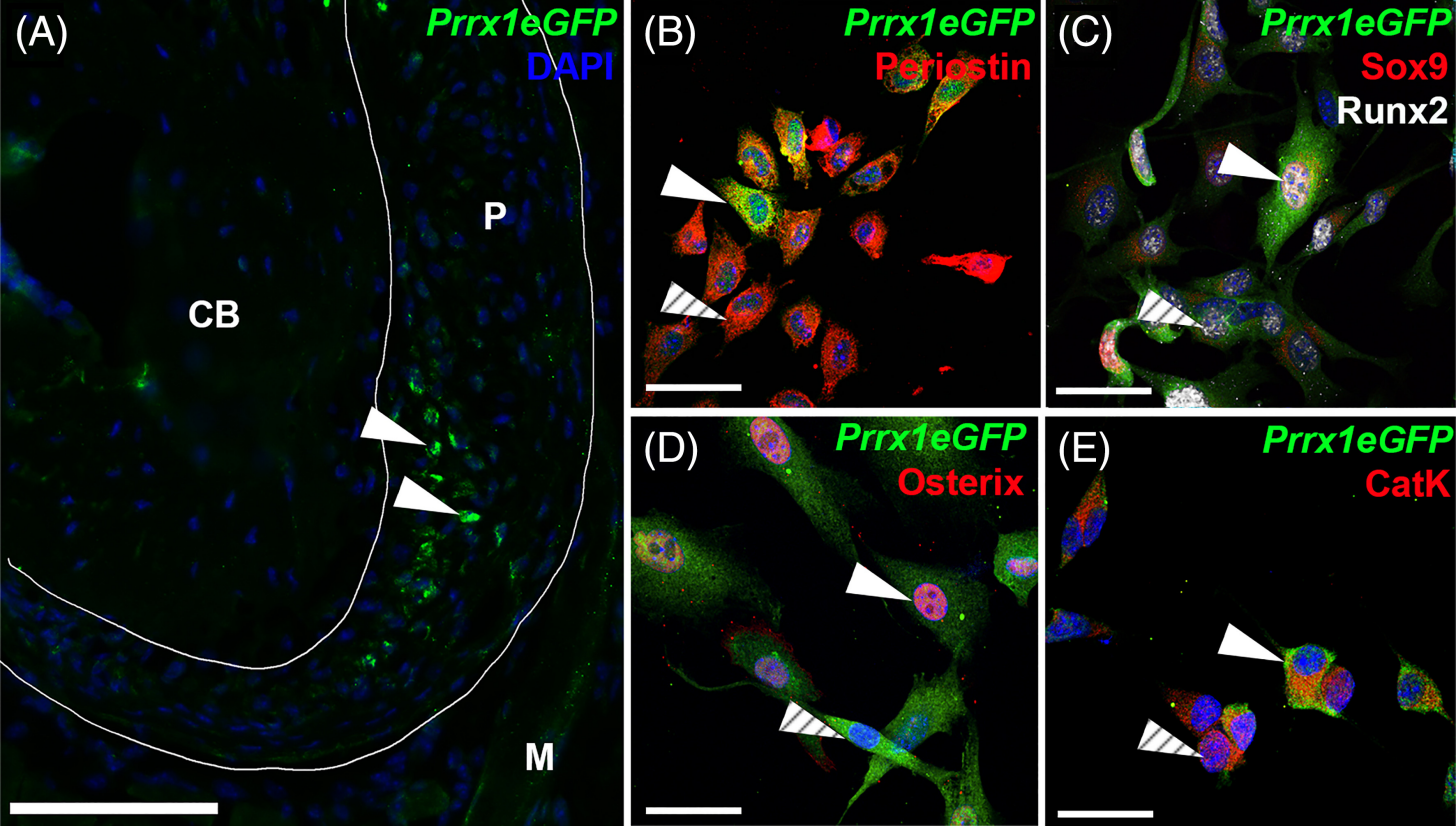
*Prrx1eGFP* labels a heterogeneous subpopulation of osteogenic periosteal cells. (*A*) *Prrx1eGFP*‐positive cells are located within the periosteum of the adult 6‐week‐old tibia, 40× magnification. Scale bar = 75 μm. (*B*) Isolated periosteal cells express periostin in vitro, a subpopulation of which are GFP‐positive (solid arrowhead indicates GFP‐positive cell, dashed arrowhead indicates GFP‐negative cell). (*C*) GFP‐positive cells can express either Sox9 or Runx2 alone (dashed arrowhead) and possible bipotential GFP‐positive cells that express both Sox9 and Runx2 (solid arrowhead). (*D*) Osterix is expressed in a subpopulation of GFP‐positive periosteal cells (solid arrowhead; dashed arrowhead indicates GFP‐labeled cells negative for Osterix). (E) Cathepsin K is expressed in both GFP‐positive (*E*, solid arrowhead) and GFP‐negative (dashed arrowhead) cells of the periosteum, 60× magnification. Scale bar = 50 μm.

We found that hematoxylin Van Gieson resolves the inner cambium layer very effectively, which is more cellular and stains yellow/brown, from the outer fibrous layer, which contains fewer cells and stains red/pink (Fig. 3*A*, *C*, *E*). Labeling serial sections with the periosteal extracellular matrix protein Postn (Supplemental Fig. S3*C*) confirms this region is periosteum. Immunohistological staining (Fig. 4*C*, *G*, *K*) in combination with serial section hematoxylin Van Gieson staining (Fig. 3*A*, *C*, *E*) confirms the presence of GFP‐positive cells within the inner cambium layer, as previously described in a different *Prrx1* transgenic line.^(^ ^25^ ^)^ In contrast to this earlier study, however, we also detect GFP‐positive cells within the outer fibrous layer (Fig. 3 *D*, black arrowheads). Interestingly, we also observe some GFP‐positive cells at the inner boundary of the cambium layer apparently invading into cortical bone (Fig. 3*D*, *F*, white arrowheads).

### *Prrx1eGFP* cells in the adult periosteum can be isolated and express markers of the osteogenic and chondrogenic lineage in vitro

*Prrx1eGFP* labels a subset of cells distributed within the adult periosteum (Fig. 4*A*). To characterize these labeled cells further, we isolated the heterogeneous periosteal cell population from the hindlimbs of 6‐week‐old transgenic mice. In vitro, isolated cells express Postn, confirming they are periosteal‐derived cells (PDCs), whereas only a subset of these are GFP‐positive (Fig. 4*B*). Although we only detect a rare Sox9‐positive population of periosteal cells in the adult in vivo (data not shown) similar to that reported previously,^(^ ^40^ ^)^ interestingly, after isolation and culture in vitro, we observe Sox9 expression in a greater proportion of cells (Fig. 4*C* and Supplemental Fig. S4*B*). We observe GFP‐positive cells that express Sox9 and/or Runx2, consistent with individual GFP‐labeled cells having either chondrogenic or osteogenic potential, in addition to cells that co‐express both.

Sox9/Runx2 indicates a third population with osteochondrogenic bipotential (Fig. 4*C*). Additionally, a subpopulation of *Prrx1eGFP*‐labeled periosteal cells express Osx identifying GFP‐labeled cells that are committed to the osteogenic lineage (Fig. 4*D*). In vitro, we observe Cathepsin K staining in both *Prrx1eGFP*‐positive and ‐negative periosteal cells (Fig. 4*E*). Together, these results demonstrate the *Prrx1eGFP* labels a heterogenous cell population osteogenic and chondrogenic potential in vitro.

**Fig. 5:**
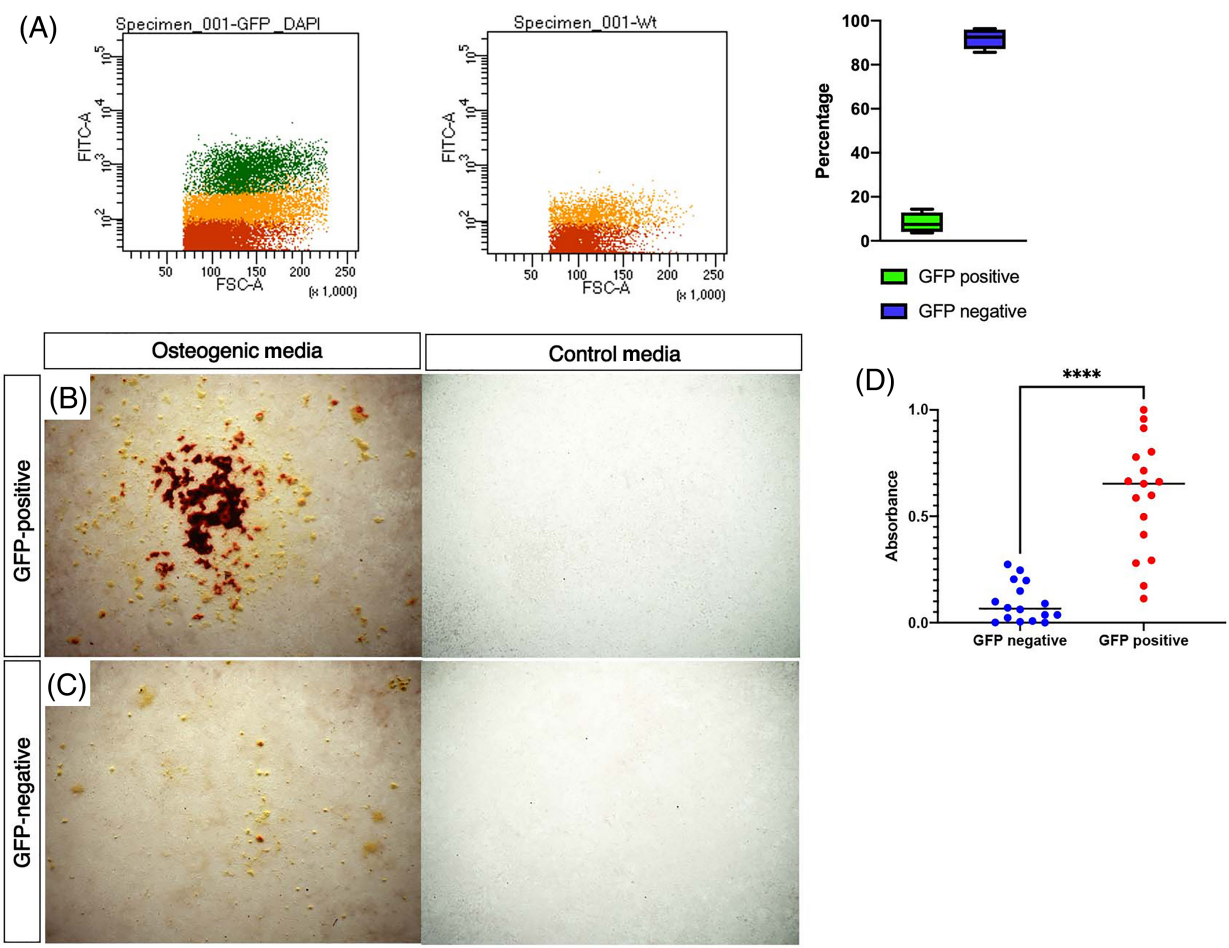
The osteogenic potential of the *Prrx1eGFP*‐labeled subpopulation of periosteal cells is greater than the GFP‐negative population. (*A*) FACS scatter plots of pooled freshly isolated periosteal cells isolated from 5× *Prrx1eGFP* mice and 1× WT control, cells isolated on the same day. Wild‐type (WT) periosteal samples demonstrate a degree of autofluorescence indicated by the orange dots (red dots show no fluorescence). GFP‐positive cells are indicated with the green dots. An average, 8.25% (*n* = 5) of live cells isolated from the periosteum are *Prrx1eGFP*‐positive. (*B, C*) Osteogenic assays stained with Alizarin Red to detect mineralized nodules. Bright‐field images of *Prrx1eGFP*‐labeled PDCs (*B*), GFP‐negative PDCs (*C*) after 21 days in either osteogenic or control media. 2.5× magnification. (*D*) Quantification of Alizarin Red staining indicating GFP‐positive cells are significantly more osteogenic than GFP‐negative cells. Data normalized, with outliers removed (*n* = 7), GFP‐negative *n* = 16, GFP‐positive *n* = 17, unpaired *t* test, ****p < 0.001.

Tissue‐resident stem cells generally constitute a minor proportion of the total cell population within different tissues; for example, approximately 4.8% to 5.8% of muscle fiber nuclei in rat and mouse are the resident stem cells.^(^ ^41^ ^)^ Using FACS to enrich for GFP‐positive and GFP‐negative cell populations (Fig. 5*A*), we find, on average, 8.25% (*n* = 5) of live cells isolated from the periosteum are *Prrx1eGFP*‐positive. These numbers are consistent with the hypothesis that these *Prrx1eGFP*‐positive cells are labeling the resident, bipotential stem cell population in the adult periosteum with some evidence of GFP labeling in daughter cells that have committed to either osteogenic or chondrogenic lineages. Consistent with *Prrx1eGFP* labeling an osteogenic progenitor population within the periosteum, we find in osteogenic assays, the GFP‐labeled subpopulation of periosteal cells has significantly greater osteogenic potential than the unlabeled GFP‐negative cells (Fig. 5*B–D*).

### Isolated cells from adult periosteum display a loss in osteogenic potential during in vitro passage‐associated aging

**Fig. 6:**
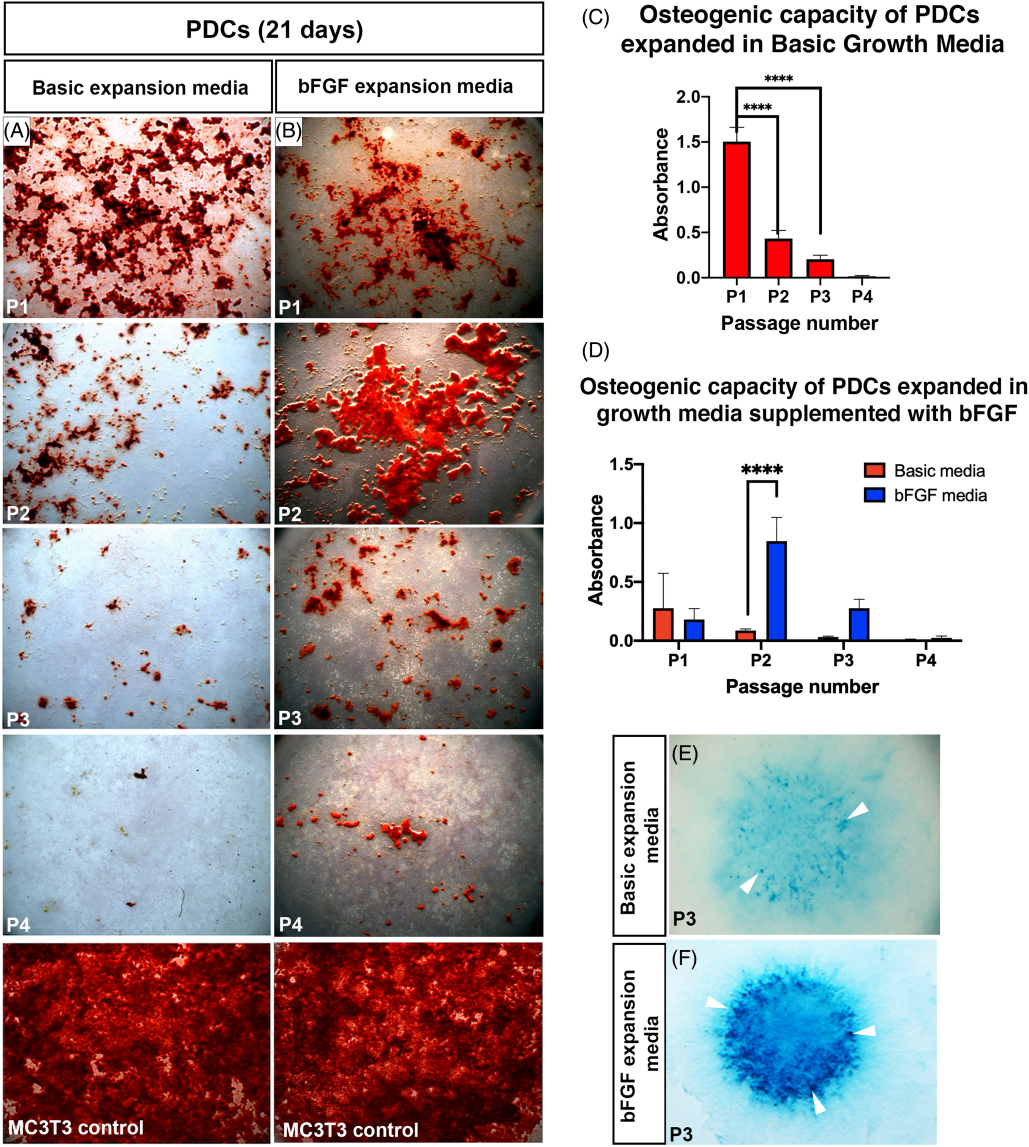
Potential of isolated heterogenous periosteal‐derived cells (PDCs) reduces as cells age in vitro. (*A*, *B*) Osteogenic assays stained with Alizarin Red to detect mineralized nodules after 21 days in osteogenic differentiation media showing the reduction in osteogenic potential of PDCs expanded in basic growth media (*A*) and growth media supplemented with bFGF (*B*) as passage number increases (P1 < P2 < P3 < P4). (*C*) Quantification of Alizarin Red staining of PDCs expanded in basic growth media (*A*) showing osteogenic potential decreases with passage when PDCs are expanded in basic media. (*D*) Equivalent data set comparing quantification of Alizarin Red staining of PDCs initially expanded in basic media or media supplemented with bFGF (*B*). (*E*, *F*) Chondrogenic assays stained with Alcian Blue to detect cartilage nodules (arrowheads) after 7 days in chondrogenic media showing the chondrogenic potential of PDCs expanded in basic growth media (*E*) and growth media supplemented with bFGF (*F*). Error bars represent standard deviation (SD), ****p ≤ 0.0001, *n* = 3.

We compared the osteogenic potential of PDCs that had been maintained for different periods of time in in vitro culture. Isolated, unsorted PDCs successfully form bone nodules in osteogenic culture after 21 days; however, we observe with each subsequent passage the amount of mineralization decreases, indicating the cells become progressively less osteogenic as they age in vitro (Fig. 6*A*). From passage 1 to passage 2, the extent of mineralization decreases by 73% (*p* ≤ 0.0001) (Fig. 6*C*). By passage 3, mineralization is reduced further. Therefore, in these culture conditions, PDCs lose the majority of their osteogenic capacity during the first passage. Furthermore, although the MC3T3 osteogenic cell line produces a uniform mineralized layer on the base of the culture dish, after the same period of time, the heterogeneous population of PDCs produce isolated mineralized nodules (Fig. 6*A*), suggesting only a subpopulation of cells in the PDC isolates have osteogenic capacity.

We found the addition of bFGF to the expansion media extends the osteogenic potential of isolated PDCs and enhances their osteogenic potential from the first to the second passage (Fig. 6*B*, *D*). At P1, there is no difference in the osteogenic potential of PDCs expanded in media supplemented with bFGF compared with those without. However, after two passages, PDCs expanded with the addition of bFGF show an increase in the amount of mineral deposited, demonstrating an increase in their osteogenic potential compared with their P1 counterparts. In comparison, PDCs expanded without bFGF show a decrease in mineralization between P1 and P2. By P3, the osteogenic capacity of these cells decreases; nevertheless, the presence of bFGF in the expansion media enhances the osteogenic capacity of cells compared with those expanded without. By P4, PDCs expanded without bFGF have lost their osteogenic capacity entirely, whereas those exposed to bFGF, although diminished, are still capable of forming bone in vitro.

Isolated, unsorted PDCs are also capable of forming cartilage nodules in chondrogenic culture after 7 days, and again, the addition of bFGF to the expansion media enhanced their chondrogenic potential (Fig. 6*E*, *F*).

### *Prrx1eGFP* ‐labeled cells in the adult periosteum are enriched at sites of mechanical load

Mechanical loading is integral to the regulation of bone remodeling and the maintenance of bone mass, a relationship that is based upon the “mechanostat” theory that increased load equates to higher bone mass.^(^ ^42^, ^43^ ^)^ The periosteum is sensitive to mechanical stimuli, and there is evidence to suggest that progenitor cells within the periosteum are biomechanically responsive.^(^ ^44^, ^45^ ^)^ In addition, loads applied to the skeleton are not uniformly distributed throughout the bone, with certain “hot spots” coming under more significant load than other regions. For example, muscle attachment sites with larger insertion areas distribute load over a wider area, whereas smaller attachment sites result in more focal, higher load to the underlying bone.^(^ ^39^, ^46^ ^)^

**Fig. 7:**
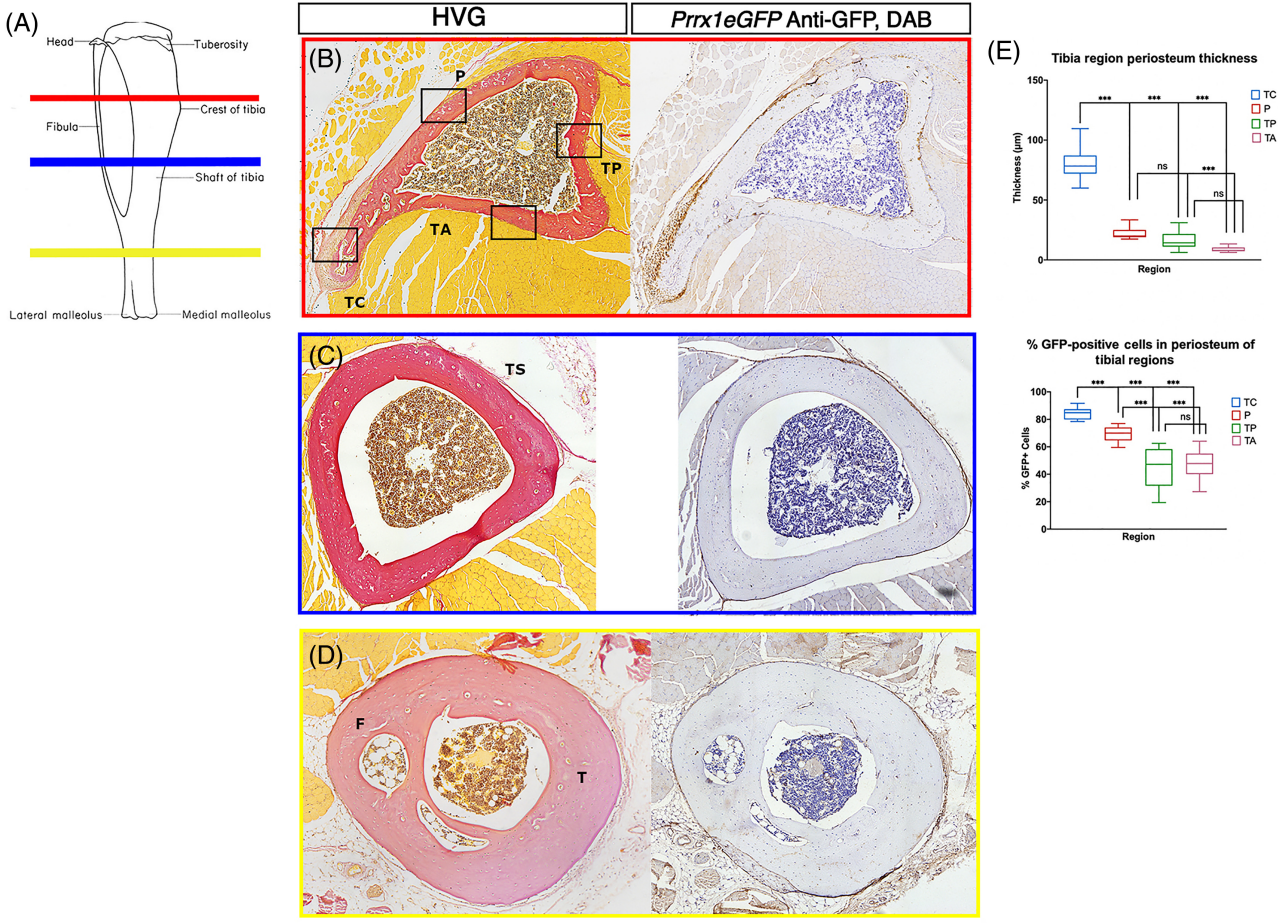
Variation in periosteal thickness and GFP‐positive cell content in the 6‐week‐old *Prrx1eGFP* tibia. (*A*) Schematic indicating the locations along the length of the tibia where serial sections were analyzed, tibial crest (red), midshaft (blue), and distal tibia (yellow). Colors correspond to boxed images in *B–D*. (*B–D*) Tile scan images of adjacent serial sections stained with HVG or anti‐GFP/DAB at the level of the tibial crest (boxed in red) (*B*), midshaft of the tibia (boxed in blue) (*C*), in a region free of muscle attachments, and distal tibia (boxed in yellow) (*D*), at the point where the fibula fuses with the tibia. (*E*) Box and whisker plots showing the varying distribution of periosteal thickness and GFP^+^ cell number in periosteum in mouse tibial regions: crest (TC), popliteus (P), posterior (TP), and anterior (TA), which correspond to the boxed regions of *B*. TS = tibial shaft; T = tibia; F = fibula.

Our initial, gross observation of the *Prrx1eGFP*‐labeled cells in the bones of the transgenic mouse indicated that these cells are distributed nonuniformly along the lengths of bones, with some regions showing a greater intensity of GFP staining. Additionally, we observed periosteal thickness varies considerably from one region to another (Fig. 7). To examine the distribution of GFP‐labeled cells in the *Prrx1eGFP* transgenic during adult stages, we analyzed transverse sections through the tibia in young adult mice (6 to 12 weeks old) (Fig. 7 and Supplemental Fig. S5). The periosteum at the tibial crest (TC; Fig. 7*B*), the site of insertion of sartorius, gracilis, and semitendinosus muscles (at the pes anserinus), is significantly thicker, in both the cambium and fibrous layers, than other regions at the same longitudinal level of the bone. This includes the posterior region of the tibia where the popliteus muscle inserts (P), the site of the tibialis posterior (TP) muscle origin, and the anterior, lateral region of the tibia, where the tibialis anterior (TA) originates (Fig. 7*B*, *E*). *Prrx1eGFP‐*labeled cells are enriched at the tibial crest, with more than 80% of the cells in the periosteum GFP‐positive. The site of the popliteus insertion is also enriched for GFP‐labeled cells (69% GFP‐positive). This compares to less than 50% GFP‐positive in regions of muscle origin (TP and TA) (*n* = 12) (Fig. 7*B*, *E* and Supplemental Fig. S5). Similar distribution of *Prrx1eGFP‐*labeled cells is observed in both the cambium and fibrous layers. At sites not associated with muscle attachment (Fig 7*B*, *C*), the number of *Prrx1eGFP‐*labeled cells found in the periosteum is much lower.

### Periosteal thickness and *Prrx1eGFP* ‐positive cell content decreases with age

**Fig. 8:**
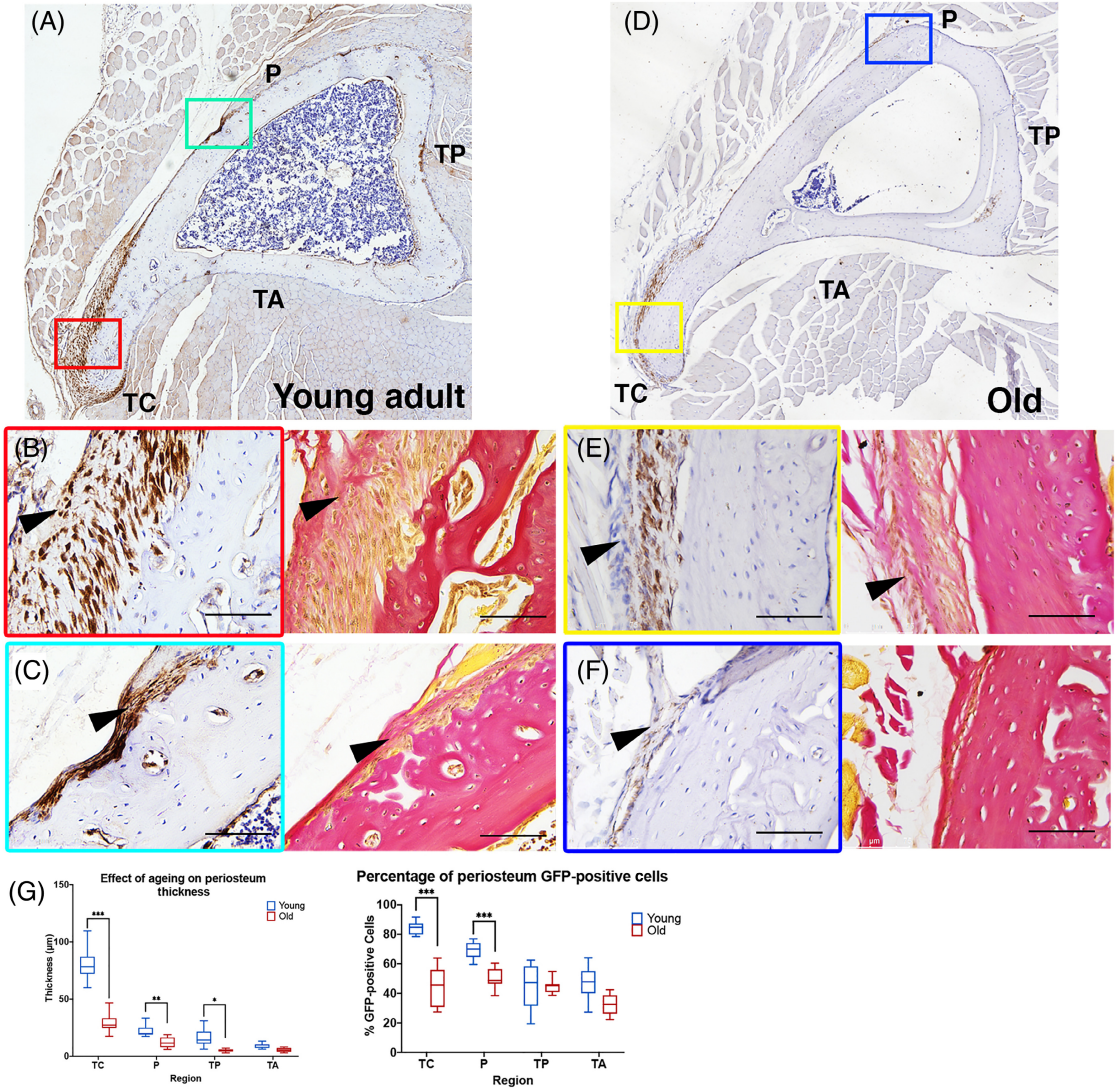
Periosteal thickness and GFP‐positive cell content decreases with age. Sections of tibia at equivalent regions of young adult (6–8 weeks) (*A*) and old (12 months) (*D*) specimens, stained for GFP (using a DAB secondary), showing *Prrx1eGFP*‐labeled cells (brown). Colored boxes outline areas shown in (*B*, *C*, *E*, *F*). Sections of tibia at equivalent regions of young adult (6–8 weeks) (*B*, *C*) and old (12 months) (*E*, *F*) specimens, stained for GFP (using a DAB secondary), showing *Prrx1eGFP*‐labeled cells (brown) and an adjacent section stained with HVG to distinguish the layers of the periosteum (40× magnification). (*G*) Quantification of periosteal thickness and GFP^+^ cell number in the whole periosteum in mouse tibial regions: crest (TC), popliteus (P), posterior (TP), and anterior (TA). Error bars represent standard deviation (SD). *p ≤ 0.03, **p ≤ 0.002, ***p ≤ 0.001. Young *n* = 12 and old *n* = 15. The *p* values were calculated using multiple *t* tests and the Holm–Sidak method.

Our analysis of isolated PDCs in culture reveals a progressive reduction in osteogenic potential with increasing time in culture (Fig. 6). To examine the effects of aging on *Prrx1eGFP*‐labeled cells in vivo, we compared equivalent transverse sections of tibia from young adult (6 to 12 weeks) and old (10 to 12 months) mice. Because we observed a wide divergence in periosteal thickness and GFP cell number at sites in the proximal tibia at the level of the tibial crest (Fig. 7), we chose this region to quantify and compare between young adult and aged mice (Fig. 8).

We observe an overall reduction in periosteal thickness and the number of GFP‐labeled cells in aged specimens across all sites tested. Moreover, the greatest reduction in thickness is found at sites where the periosteum is thickest in the young adult. For example, the largest reduction in thickness is found at the tibial crest compared with other sites, such as the popliteus insertion and TP and TA muscle origins (Fig. 8, *p* ≤ 0.001). A similar level of reduction in thickness is observed in both the cambium and fibrous layers, and this pattern is also observed when numbers of GFP‐positive cells are compared (Supplemental Fig. S6). *Prrx1eGFP‐*labeled cells are enriched in areas of muscle insertion (TC and P) compared with sites of muscle origin (TP and TA), coinciding with periosteal thickness, and this enrichment is found in both layers of the periosteum. In aged samples, areas of muscle insertion show a significant reduction in GFP‐positive cell numbers in both layers of the periosteum (*p* ≤ 0.001), whereas areas of muscle origin have only a minor decrease. The greatest loss of *Prrx1eGFP* cell enrichment is found in the tibial crest region, where the numbers of GFP‐positive cells almost halves in older mice compared with young adult mice (Fig. 8*G* and Supplemental Fig. S6). Together, these results demonstrate that *Prrx1eGFP‐*labeled cells within the periosteum are lost with age in both layers of the periosteum, and this is associated with the overall reduction in periosteal thickness.

## Discussion

Several recent studies have identified candidate markers for periosteal skeletal stem cells involved in bone homeostasis and repair;^(^ ^4^, ^15^, ^47^, ^48^ ^)^ however, there is no consensus on a definitive periosteal stem‐cell marker. Here, we report the creation of a *Prrx1eGFP* transgenic mouse that live‐labels a subpopulation of cells within the periosteum with GFP. The labeling is sufficiently robust to be seen under episcopic fluorescence and to enable isolation/enrichment of GFP‐positive cells by FACS. Immunostaining for GFP protein is required to detect *Prrx1eGFP* expression when staining fixed tissue sections, however. Previous work utilizing the *Prx1CreER‐GFP* transgenic makes use of an IRES to allow co‐expression of CreER and GFP under the same Prrx1 promoter. A common technical drawback of using IRES, however, is that expression can be low, resulting in weak GFP expression that is hard to detect both in vivo and in vitro. This can explain why in vivo analysis relied on tamoxifen‐induced cre‐recombination, with a Rosa26 LacZ reporter,^(^ ^25^ ^)^ rather than the GFP fluorescence. Labeling cells via tamoxifen‐inducible cre‐recombination is useful for cell lineage analysis but, since all daughter cells of progenitors are also labeled, it does not allow for specific labeling of the skeletal stem progenitor population alone. The live nature of our *Prrx1eGFP* transgenic allows for a more faithful examination of the association of *Prrx1*‐expressing cells within the developing periosteum, how they contribute to new bone formation, and permits their isolation by FACS for analysis of their osteogenic capacity in vitro.

It remains unclear whether the perichondrium of the appendicular skeleton originates from the same condensation of mesenchymal cells that form the cartilage anlagen in the developing limbs or from a population of cells surrounding these cartilage condensations. Lineage tracing studies using a Sox9‐Cre knock‐in transgenic indicate that Sox9‐expressing cells give rise to chondrocytes and osteoblasts in cartilage primordia and perichondrium, supporting the idea that the perichondrium is derived from cells that have expressed Sox9.^(^ ^49^ ^)^ Further studies, using a *Sox9CreERT2* and administration of tamoxifen at a range of embryonic stages, demonstrate that Sox9‐expressing cells at E10.5 contribute to the perichondrium, but at stages later than E12.5, the perichondrium is not labeled, indicating that at stages later than E12.5, periosteal precursor cells do not express Sox9.^(^ ^50^ ^)^ Consistent with these observations, by E14.5, we can identify a ring of *Prrx1eGFP*‐labeled/Sox9‐negative perichondrial cells encircling a core of Sox9‐positive chondrocytes of the cartilage anlagen, which serves as early molecular evidence of the formation of the perichondrium.

**Fig. 9:**
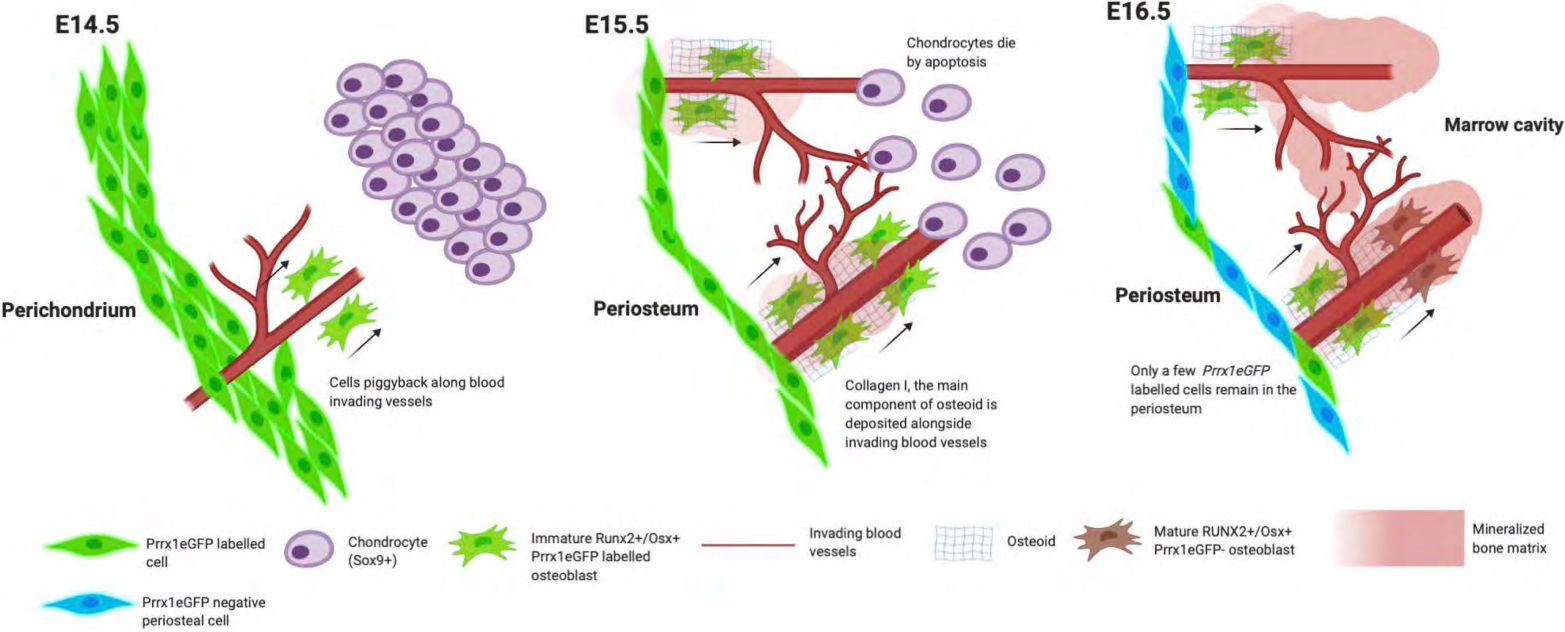
Schematic diagram showing how *Prrx1eGFP*‐labeled cells in the developing perichondrium might contribute to the bone collar. At E14.5, Sox9‐positive chondrocytes retract away from the surrounding mesenchyme and endothelial cells invade. *Prrx1eGFP* cells from the perichondrium are found in association with the endothelial cells. By E15.5, endothelial cells organize into mature blood vessels, and *Prrx1eGFP* cells piggyback along invading blood vessels, depositing bone ECM (collagen I) as they differentiate along the osteoblast lineage. This gradually fills the space between the perichondrium and cartilage with bone, resulting in the formation of the periosteum by E15.5. By E16.5, only a few *Prrx1eGFP*‐labeled cells remain in the periosteum.

Serial staining for *Prrx1eGFP*, Sox9, Runx2, Osx, and PECAM in tibia between E14.5–E16.5 demonstrates the restriction of the *Prrx1eGFP* label to the perichondrium and suggests cells of the perichondrium contribute to formation of the bone collar. At E14.5, we observe endothelial cells invading the space between the condensed cartilage and the perichondrium. Previous work has demonstrated immature osteoblast precursors piggyback on vascular endothelial cells that extend from the perichondrium to the hypertrophic cartilage^(^ ^51^ ^)^ and secrete type I collagen, the main component of osteoid, and cover the surface of forming blood vessels.^(^ ^37^ ^)^ Consistent with these observations, we see *Prrx1eGFP*‐labeled perichondrial cells with osteogenic potential (as indicated by Runx2 and Osterix expression in serial sections) use these vascular cells as a scaffold to bridge the gap between the perichondrium and presumably depositing bone matrix as they do so. This marks the transition of the perichondrium (around cartilage) into the periosteum (around bone) and the formation of the bone collar at E15.5. This suggests that at least some osteoblasts contributing to formation of cortical bone originate from *Prrx1eGFP*‐positive cells within the perichondrium (Fig. 9). However, lineage tracing experiments would be required to definitively prove this hypothesis.

Previous studies using a Cathepsin K‐Cre reporter system (*Ctsk‐cre; mTmG*) have suggested Cathepsin K could be a marker of periosteal stem cells because of their apparent capacity for self‐renewal and their ability to differentiate into mature osteoblasts, adipocytes, and chondrocytes in vitro and form bone in vivo.^(^ ^15^ ^)^ However, we find no overlap in the *Prrx1eGFP‐*labeled and Cathepsin K‐positive periosteal populations in vivo (Supplemental Fig. S1), and their location close to the bone surface suggests that the Cathepsin K‐positive cells may be a more committed population. Cathepsin K is an enzyme involved in the breakdown of collagen matrix,^(^ ^52^ ^)^ and it seems unlikely a quiescent stem cell would produce an enzyme that could destroy its niche. Instead, it is possible that the cells expressing this marker are progeny escaping the confines of the niche microenvironment. Again, lineage tracing experiments will be required to definitively prove this hypothesis.

Under normal homeostatic conditions in vivo, cells of the periosteum contribute to appositional bone growth, demonstrating their osteogenic capacity. It is only under fracture repair conditions that periosteal cells show chondrogenic capacity in vivo.^(^ ^53^ ^)^ A rare Sox9‐positive osteochondroprogenitor population has been reported in the periosteum of adult bone, which is mobilized during fracture repair.^(^ ^40^ ^)^ In our IHC studies, we also detect a rare population of Sox9‐postive cells within the adult periosteum in vivo (data not shown). Isolated PDCs are highly heterogenous with different subpopulations expressing *Prrx1eGFP*, Sox9, Runx2, and/or Osterix, suggesting the periosteum contains a mixture of bipotential, unipotential, and committed cells. The in vitro expression of Sox9 suggests isolation of these cells from their normal environment elicits a fracture‐like response, with an upregulation in Sox9 expression permitting cells to become chondrogenic. In in vitro culture, we found a subset of *Prrx1eGFP*‐labeled cells express both Sox9 and Runx2, suggesting the *Prrx1eGFP* transgene is labeling bipotential progenitors within the periosteum. In agreement with the hypothesis that in vitro culture is analogous to a fracture environment, we found Cathepsin K within a subpopulation of *Prrx1eGFP*‐labeled cells. As previously stated, this could indicate Cathepsin K marks cells immediately leaving the stem cell niche, post fracture, whereas in vivo *Prrx1eGFP* also labels a Sox9/Runx2‐negative progenitor that may represent periosteal stem cells, given their location in vivo.

Previous studies using a *Prrx1creER‐IRES‐*GFP have shown that the GFP‐labeled population is osteogenic, but this was not compared with the unlabeled population.^(^ ^25^ ^)^ More recent studies using the same transgenic line demonstrate the GFP‐labeled population has enhanced osteogenic potential compared with a CD90‐positive/GFP‐negative subpopulation.^(^ ^54^ ^)^ Additionally, because of the use of an IRES reporter in these studies,^(^ ^25^, ^54^ ^)^ it is likely the GFP‐positive cells isolated by FACS are selecting for a smaller population, and it is probable that some GFP‐negative periosteal cells are *Prrx1creER‐IRES‐*GFP‐positive but with GFP expression that is too low to detect. The *Prrx1eGFP* transgenic mouse reported here, however, is a live label of the Prrx1 enhancer that does not include an IRES and therefore appears brighter. This likely allows for a more faithful isolation by FACS and downstream analysis of the utility of the Prrx1 enhancer for labeling an osteogenic population. We have herein compared the osteogenic potential of the live‐labeled GFP‐positive and GFP‐negative populations in the *Prrx1eGFP* transgenic and show that the vast majority of osteogenic cells in the periosteum are within the GFP‐positive population. This demonstrates that *Prrx1eGFP* can enrich for the osteogenic progenitor population of the periosteum, a subset of which would be predicted to the periosteal stem cell.

We next examined the capacity of the heterogeneous PDC population in vitro and found PDCs were capable of forming both bone and cartilage (Fig. 6), which is in line with our previous studies on human and mouse periosteal cells.^(^ ^5^, ^55^ ^)^ Interestingly, as PDCs were expanded in vitro, their osteogenic potential reduced. Previous studies on the expansion of murine periosteal progenitor cells have reported the presence of bFGF increases the number of colonies with osteogenic potential and the expression of skeletal stem cell markers.^(^ ^56^ ^)^ Other in vitro studies have shown FGF signaling stimulates osteoblast proliferation, differentiation, and synthesis of osteocalcin in the long term.^(^ ^57^, ^58^, ^59^ ^)^ However, in short‐term cultures, bFGF initially reduces osteocalcin synthesis.^(^ ^60^ ^)^ This could explain why we see an initial increase in osteogenic potential when we expand PDCs in media supplemented with bFGF compared with those without but subsequently see this reduction with further expansion. It is possible FGF selects for a more osteogenic subpopulation, perhaps periosteal stem cells, thus extending the capability of these cells to produce mineralized bone matrix.

*Prrx1eGFP*‐labeled cells are enriched at sites of muscle insertion, within tibia most notably at the tibial crest (Figs. 7 and 8). This *Prrx1eGFP* enrichment coincides with an increase in the thickness of both the fibrous and cambium layers. The pattern of periostin staining confirms this thickened region is periosteum and not an enthesis. These results suggest that the *Prrx1eGFP*‐labeled cells at the sites of enrichment are a biomechanically responsive, progenitor population involved in bone homeostasis that increase bone deposition in response to mechanical load. Knowledge of sites of enrichment of the progenitor cells involved in repair could be of great clinical significance for surgical treatments that involve periosteal transplantation, for example, fracture non‐unions.^(^ ^61^, ^62^, ^63^ ^)^

The live nature of the *Prrx1eGFP* transgene enables longitudinal studies to investigate how its distribution in the periosteum changes with age. We confirm the robust nature of this transgene as expression is still evident in both layers of the periosteum in 12‐month‐old animals. The periosteum becomes thinner with age.^(^ ^64^ ^)^ We found the number of *Prrx1eGFP*‐labeled cells reduces with age, most notably in the regions that show the greatest enrichment in the young adult, at sites of muscle insertion. We cannot yet distinguish whether this reduction in cells is because of a progenitor pool being progressively depleted during the life course or these populations becoming progressively less responsive or less exposed to stimulatory mechanical cues. To our knowledge, this is the first time that the association of a specific subpopulation of cells to periosteum formation has been investigated in this depth, with assessment of how the population changes with aging both in vivo and in vitro. The loss of osteogenic capacity we observe in vivo appears to mimic what we observe in vitro and has implications for age‐related diseases such as osteoporosis, where bone fragility is partially attributable to a reduction in periosteal bone formation.^(^ ^65^ ^)^

Taken together, our results indicate that the *Prrx1eGFP* transgenic labels a possible bipotential osteochondroprogenitor population with several features consistent with them being potential periosteal stem cells.

## Author Contributions

**Sarah Brown:** Conceptualization; formal analysis; investigation; methodology; validation; writing – original draft; writing – review and editing. **Saif Malik:** Formal analysis. **Maria Aljammal:** Formal analysis. **Aine O'Flynn:** Formal analysis. **Carl Hobbs:** Methodology. **Mittal Shah:** Resources. **Scott j Roberts:** Conceptualization; funding acquisition; methodology; project administration; supervision; writing – review and editing. **Malcolm Logan:** Conceptualization; formal analysis; funding acquisition; project administration; resources; supervision; writing – original draft; writing – review and editing.

## Conflict of Interest

The authors have no conflicts of interest to declare.

### Peer Review

The peer review history for this article is available at https://publons.com/publon/10.1002/jbm4.10707.

## Supporting information


**Supplemental Fig. S1.** *Prrx1eGFP* expression in the growth plate and articular cartilage. (*A*) Longitudinal section of 6‐week‐old *Prrx1eGFP* tibia showing expression in the periosteum (white arrowhead). (*B*) Minimal *Prrx1eGFP* expression is detected in the growth plate at 6 weeks of age. (*C*) Longitudinal section of E16.5 tibia showing *Prrx1eGFP* (green), Sox9 (red), and Runx2 (white) expression in the growth plate and diaphysis. *Prrx1eGFP* expression is found in a small population of cells in the both the proliferative zone (*D*) and hypertrophic zone (*E*) of the growth plate at this stage. BM = bone marrow; SB = subchondral bone; P = periosteum; GP = growth plate.

**Supplemental Fig. S2.** Periostin is not a reliable marker of the developing periosteum in the embryo. (*A–C*) HVG‐stained serial section of E14.5, E15.5, and E16.5 hindlimbs showing the development of the perichondrium (PC) and periosteum (PO). (*D–F*) Periostin expression is found in the surrounding mesenchyme at E14.5 but not in the perichondrium. Periostin is found labeling the periosteum from E15.5 onward. (*G*) Section stained with Cathepsin K (CatK) and GFP. Cathepsin K expression does not overlap with the GFP population at E16.5, 40× magnification, 25 μm scale bar. PO = periosteum; HC = hypertrophic chondrocytes; PO = periosteum; B = bone; MC = marrow cavity.

**Supplemental Fig. S3.** Comparative analysis with histological stains identify the location of *Prrx1eGFP*‐labeled periosteal cells. Serial cryosections of 6‐week‐old mouse hindlimbs from the same series shown in Fig. 3, stained alternately with hematoxylin and eosin (*A*, *C*, *E*) or toluidine blue (*B*, *D*, *F*). Serial tile scan images stained alternately with DAPI and periostin (*G*) and HVG (*H*). Periostin is restricted to both layers of the periosteum surrounding the entire periphery of a 6‐week‐old tibia, 400 μm scale bar. F = fibrous; C = cambium layers; CB = cortical bone.

**Supplemental Fig. S4.** Isolated periosteal cells elicit a fracture like response in vitro. (*A*) Image of a 6‐week‐old *Prrx1eGFP* mouse tibia at the level of the tibial crest showing an absence of Sox9 staining in the periosteum and an isolated number of GFP‐positive cells (100× magnification, 20 μm scale bar). (*B*) Isolated PDCs expanded in vitro for 96 hours and stained with Sox9 (red), *Prrx1eGFP* (green), and DAPI (blue) showing Sox9 in the nucleus of GFP‐labeled cells (solid arrowheads). CB = cortical bone; M = muscle; PC = perichondrium.

**Supplemental Fig. S5.** Quantification of periosteal thickness and GFP‐positive cell content in the 6‐week‐old *Prrx1eGFP* tibia. Box and whisker plots showing the varying distribution of periosteal thickness and GFP^+^ cell number in the cambium layer (*A*, *B*) and the fibrous layer (*C*, *D*) in mouse tibial regions: tibial crest (TC), popliteus (P), posterior (TP), and anterior (TA). Error bars represent standard deviation (SD). *p ≤ 0.03, **p ≤ 0.002, ***p ≤ 0.001, *n* = 12. The *p* values were calculated using multiple *t* tests and the Holm–Sidak method.

**Supplemental Fig. S6.** Periosteal thickness and GFP‐positive cell content decreases with age. (*A–D*) Box and whisker plots of the quantification of periosteal thickness and GFP^+^ cell number in the whole cambium (*A*, *B*) and the fibrous layer (*C*, *D*) in mouse tibial regions: tibial crest (TC), popliteus (P), posterior (TP), and anterior (TA). Regions analyzed are shown in Fig. 8. Error bars represent standard deviation (SD). *p ≤ 0.03, **p ≤ 0.002, ***p ≤ 0.001. Young *n* = 12 and Old *n* = 15. The *p* values were calculated using multiple *t* tests and the Holm–Sidak method.
