# Supplementary figures and images for: The Prrx1eGFP Mouse Labels the Periosteum During Development and a Subpopulation of Osteogenic Periosteal Cells in the Adult
Source: JBMR Plus. 2022 Dec 14;7(2):e10707. doi: 10.1002/jbm4.10707 (PMC9893263; doi:10.1002/jbm4.10707)

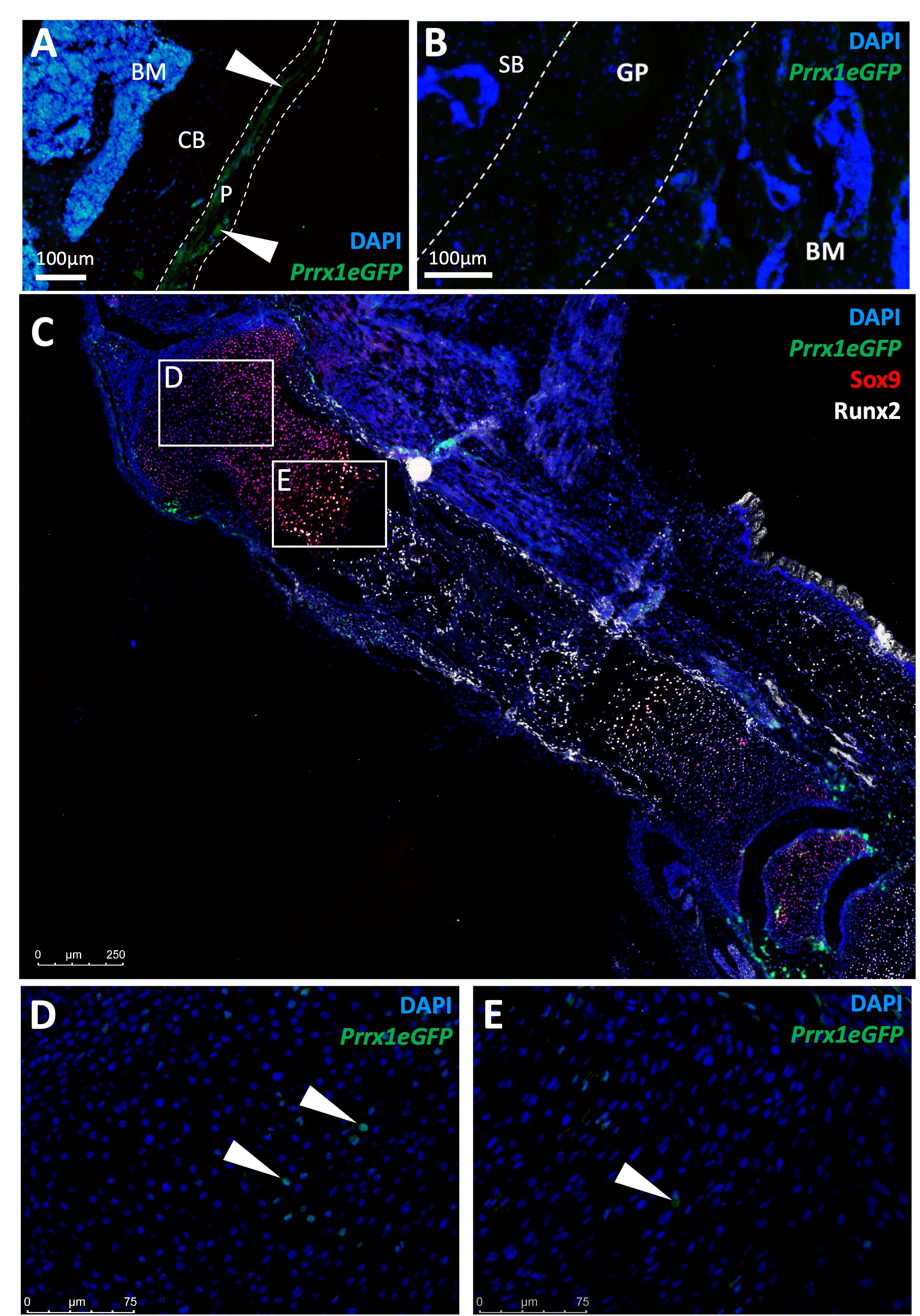

Supplement: Supplementary file 1 — Supplemental Fig. S1. Prrx1eGFP expression in the growth plate and articular cartilage. (A) Longitudinal section of 6‐week‐old Prrx1eGFP tibia showing expression in the periosteum (white arrowhead). (B) Minimal Prrx1eGFP expression is detected in the growth plate at 6 weeks of age. (C) Longitudinal section of E16.5 tibia showing Prrx1eGFP (green), Sox9 (red), and Runx2 (white) expression in the growth plate and diaphysis. Prrx1eGFP expression is found in a small population of cells in the both the proliferative zone (D) and hypertrophic zone (E) of the growth plate at this stage. BM = bone marrow; SB = subchondral bone; P = periosteum; GP = growth plate. [file JBM4-7-e10707-s002.jpg]

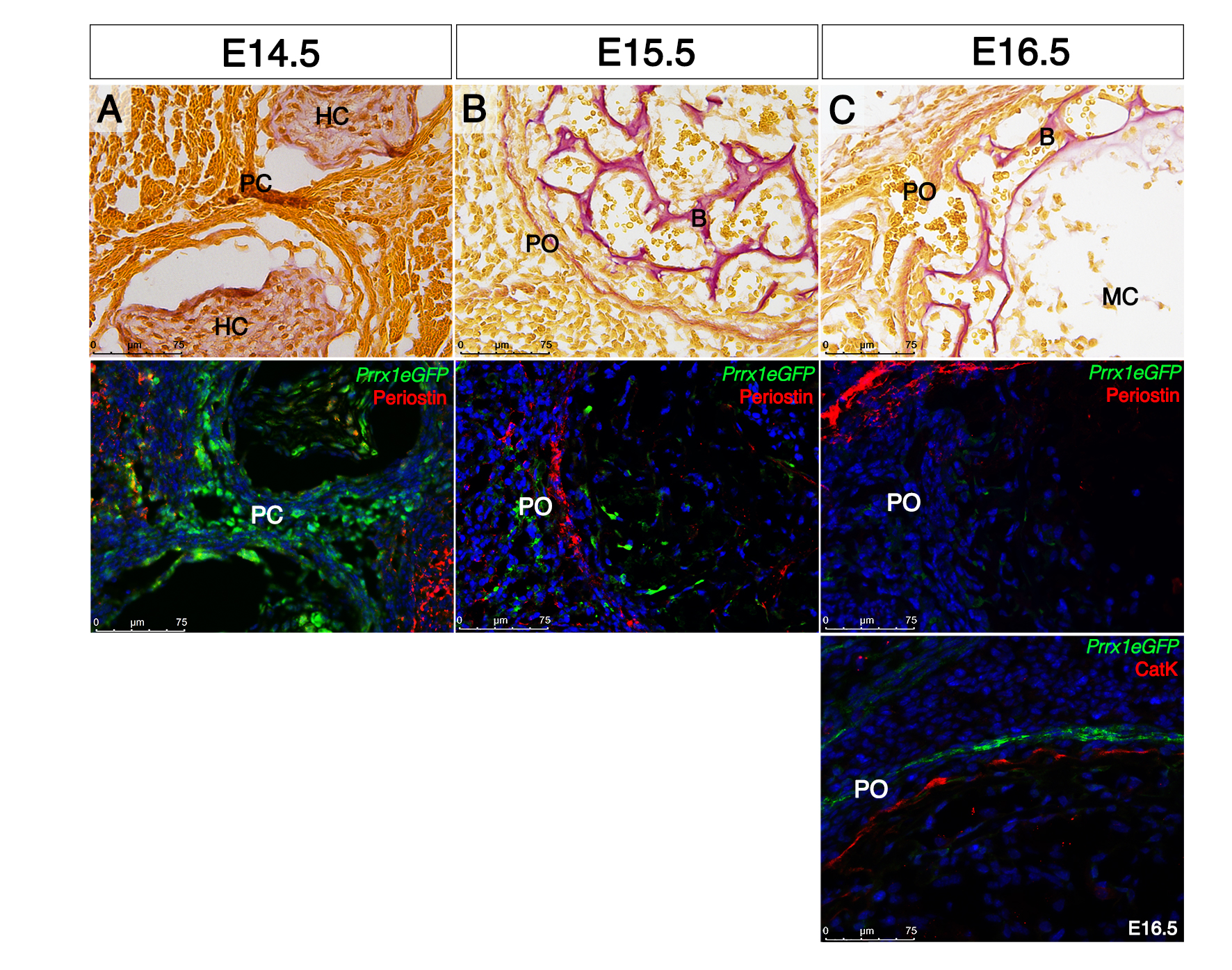

Supplement: Supplementary file 2 — Supplemental Fig. S2. Periostin is not a reliable marker of the developing periosteum in the embryo. (A–C) HVG‐stained serial section of E14.5, E15.5, and E16.5 hindlimbs showing the development of the perichondrium (PC) and periosteum (PO). (D–F) Periostin expression is found in the surrounding mesenchyme at E14.5 but not in the perichondrium. Periostin is found labeling the periosteum from E15.5 onward. (G) Section stained with Cathepsin K (CatK) and GFP. Cathepsin K expression does not overlap with the GFP population at E16.5, 40× magnification, 25 μm scale bar. PO = periosteum; HC = hypertrophic chondrocytes; PO = periosteum; B = bone; MC = marrow cavity. [file JBM4-7-e10707-s003.jpg]

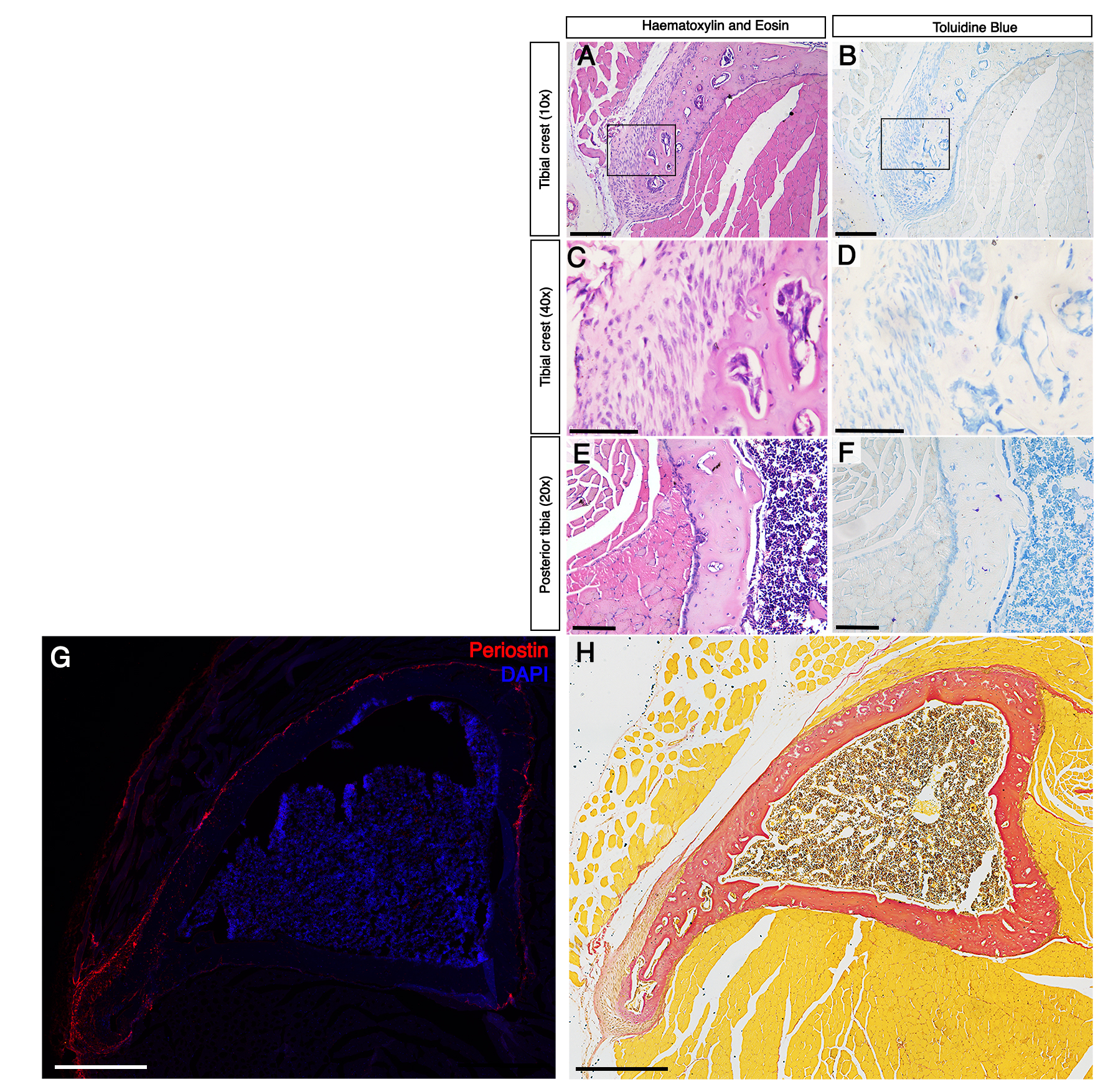

Supplement: Supplementary file 3 — Supplemental Fig. S3. Comparative analysis with histological stains identify the location of Prrx1eGFP‐labeled periosteal cells. Serial cryosections of 6‐week‐old mouse hindlimbs from the same series shown in Fig. 3, stained alternately with hematoxylin and eosin (A, C, E) or toluidine blue (B, D, F). Serial tile scan images stained alternately with DAPI and periostin (G) and HVG (H). Periostin is restricted to both layers of the periosteum surrounding the entire periphery of a 6‐week‐old tibia, 400 μm scale bar. F = fibrous; C = cambium layers; CB = cortical bone. [file JBM4-7-e10707-s005.jpg]

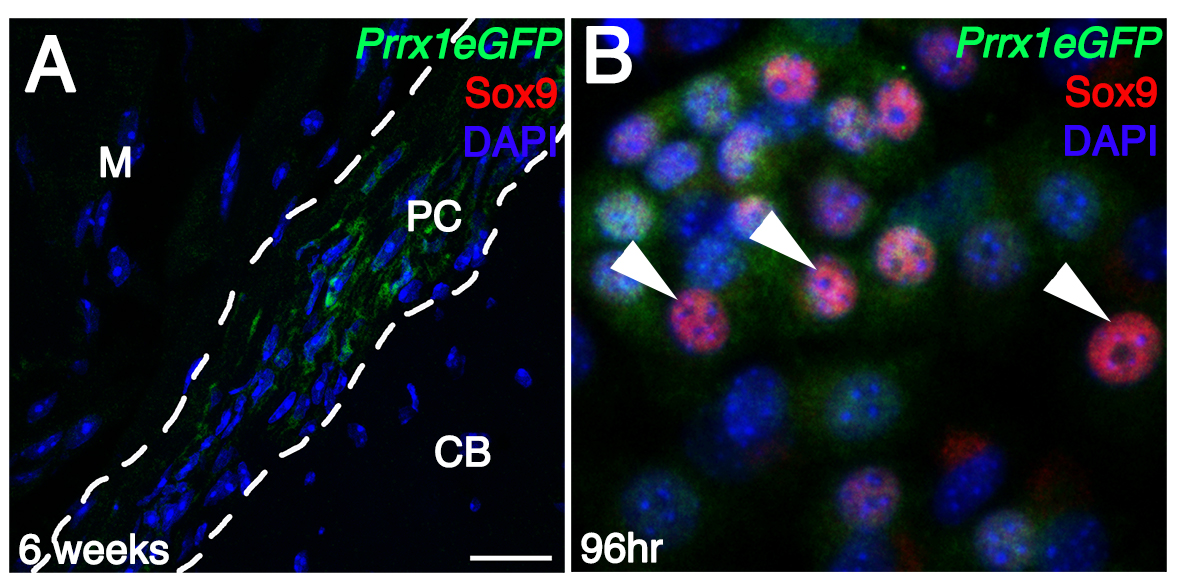

Supplement: Supplementary file 4 — Supplemental Fig. S4. Isolated periosteal cells elicit a fracture like response in vitro. (A) Image of a 6‐week‐old Prrx1eGFP mouse tibia at the level of the tibial crest showing an absence of Sox9 staining in the periosteum and an isolated number of GFP‐positive cells (100× magnification, 20 μm scale bar). (B) Isolated PDCs expanded in vitro for 96 hours and stained with Sox9 (red), Prrx1eGFP (green), and DAPI (blue) showing Sox9 in the nucleus of GFP‐labeled cells (solid arrowheads). CB = cortical bone; M = muscle; PC = perichondrium. [file JBM4-7-e10707-s004.jpg]

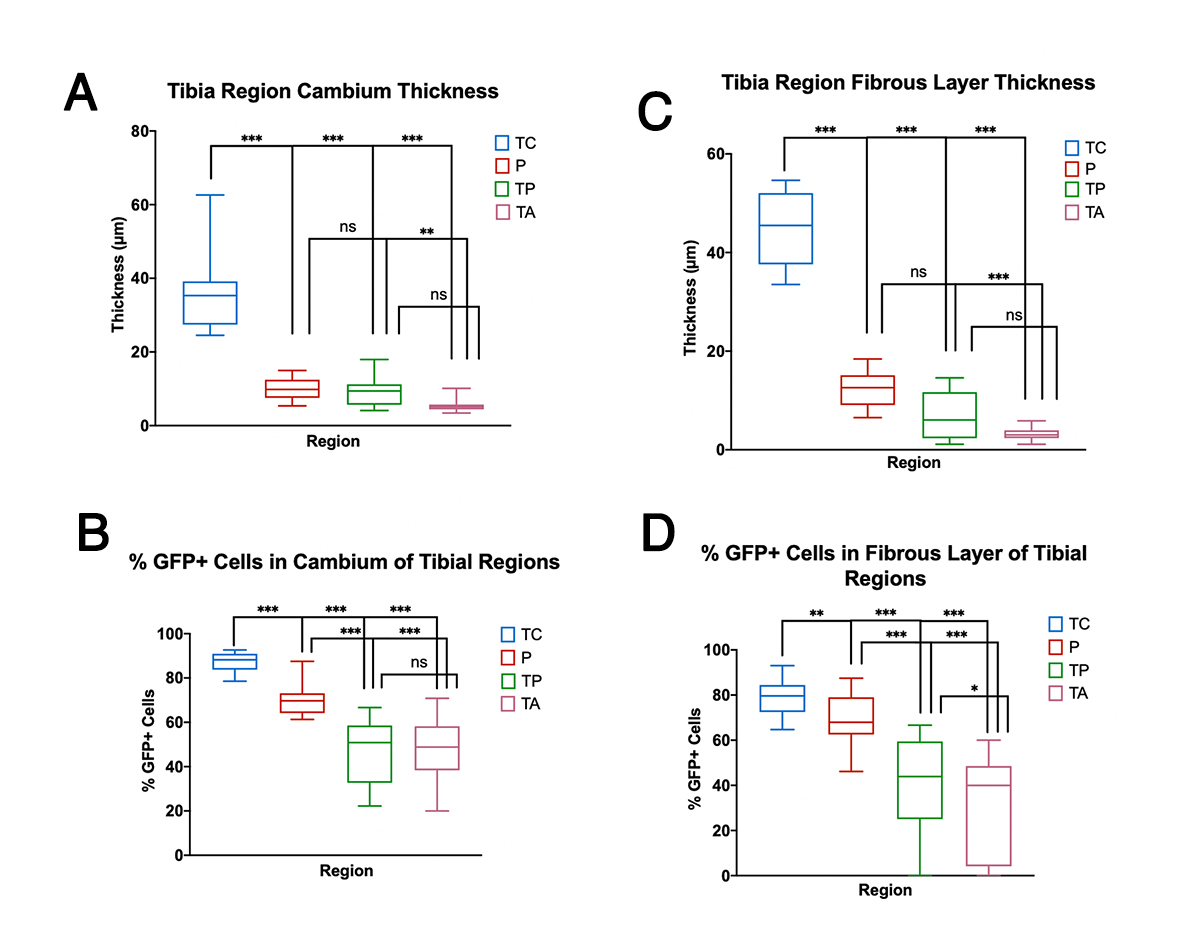

Supplement: Supplementary file 5 — Supplemental Fig. S5. Quantification of periosteal thickness and GFP‐positive cell content in the 6‐week‐old Prrx1eGFP tibia. Box and whisker plots showing the varying distribution of periosteal thickness and GFP+ cell number in the cambium layer (A, B) and the fibrous layer (C, D) in mouse tibial regions: tibial crest (TC), popliteus (P), posterior (TP), and anterior (TA). Error bars represent standard deviation (SD). *p ≤ 0.03, **p ≤ 0.002, ***p ≤ 0.001, n = 12. The p values were calculated using multiple t tests and the Holm–Sidak method. [file JBM4-7-e10707-s001.jpg]

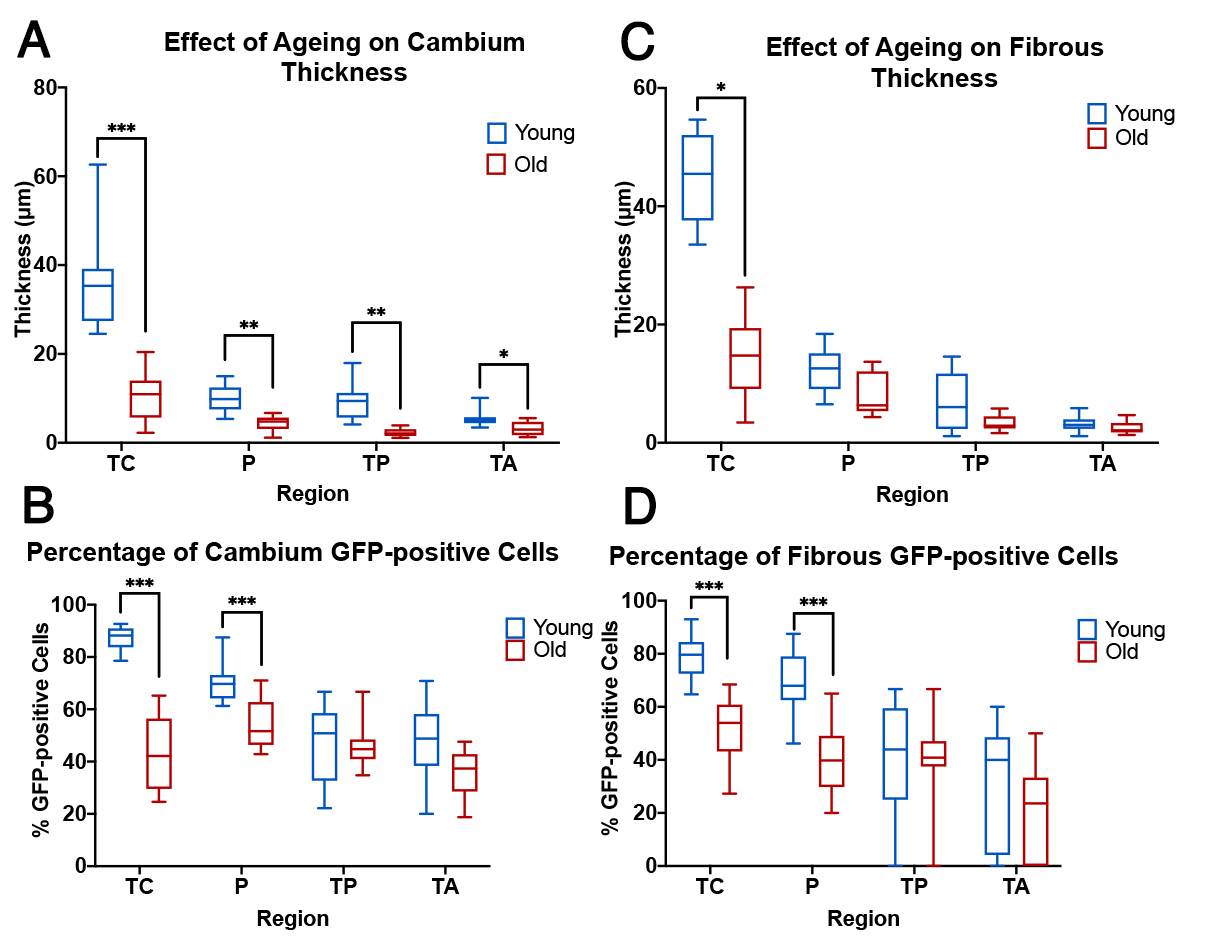

Supplement: Supplementary file 6 — Supplemental Fig. S6. Periosteal thickness and GFP‐positive cell content decreases with age. (A–D) Box and whisker plots of the quantification of periosteal thickness and GFP+ cell number in the whole cambium (A, B) and the fibrous layer (C, D) in mouse tibial regions: tibial crest (TC), popliteus (P), posterior (TP), and anterior (TA). Regions analyzed are shown in Fig. 8. Error bars represent standard deviation (SD). *p ≤ 0.03, **p ≤ 0.002, ***p ≤ 0.001. Young n = 12 and Old n = 15. The p values were calculated using multiple t tests and the Holm–Sidak method. [file JBM4-7-e10707-s006.jpg]
